# A randomized study of intensified antiretroviral treatment monitoring versus standard-of-care for prevention of drug resistance and antiretroviral treatment switch

**DOI:** 10.1097/QAD.0000000000003349

**Published:** 2022-10-04

**Authors:** Lucas E. Hermans, Rob Ter Heine, Rob Schuurman, Hugo A. Tempelman, David M. Burger, Sigrid C.J.M. Vervoort, Walter L.J.M. Deville, Dorien De Jong, Willem D.F. Venter, Monique Nijhuis, Annemarie M.J. Wensing

**Affiliations:** a Virology, Department of Medical Microbiology, University Medical Center Utrecht (UMCU), Utrecht, the Netherlands; b Ezintsha, University of Witwatersrand, Johannesburg; c Infectious Diseases & HIV Medicine, Department of Medicine, University of Cape Town, Cape Town, South Africa; d Department of Pharmacy, Radboud Institute for Health Sciences, Radboud University Medical Center, Nijmegen, the Netherlands; e Ndlovu Research Consortium, Elandsdoorn, South Africa; f Nursing Service; g Julius Global Health, The Julius Center for Health Sciences and Primary Care, University Medical Center Utrecht, the Netherlands; h HIV Pathogenesis Research Unit, Faculty of Health Sciences, University of Witwatersrand, Johannesburg, South Africa.

**Keywords:** antiretroviral treatment, drug exposure testing, drug resistance, viral load monitoring

## Abstract

**Introduction:**

Standard-of-care antiretroviral treatment (ART) monitoring in low and middle-income countries consists of annual determination of HIV-RNA viral load with confirmatory viral load testing in case of viral rebound. We evaluated an intensified monitoring strategy of three-monthly viral load testing with additional drug exposure and drug resistance testing in case of viral rebound.

**Methods:**

We performed an open-label randomized controlled trial (RCT) at a rural South African healthcare clinic, enrolling adults already receiving or newly initiating first-line ART. During 96 weeks follow-up, intervention participants received three-monthly viral load testing and sequential point-of-care drug exposure testing and DBS-based drug resistance testing in case of rebound above 1000 copies/ml. Control participants received standard-of-care monitoring according to the WHO guidelines.

**Results:**

Five hundred one participants were included, of whom 416 (83.0%) were randomized at 24 weeks. Four hundred one participants were available for intention-to-treat analysis. Viral rebound occurred in 9.0% (18/199) of intervention participants and in 11.9% (24/202) of controls (*P* = 0.445). Time to detection of rebound was 375 days [interquartile range (IQR): 348–515] in intervention participants and 360 days [IQR: 338–464] in controls [hazard ratio: 0.88 (95% confidence interval (95% CI): 0.46–1.66]; *P* = 0.683]. Duration of viral rebound was 87 days [IQR: 70–110] in intervention participants and 101 days [IQR: 78–213] in controls (*P* = 0.423). In the control arm, three patients with confirmed failure were switched to second-line ART. In the intervention arm, of three patients with confirmed failure, switch could initially be avoided in two cases.

**Conclusion:**

Three-monthly viral load testing did not significantly reduce the duration of viraemia when compared with standard-of-care annual viral load testing, providing randomized trial evidence in support of annual viral load monitoring.

## Introduction

The roll-out of antiretroviral treatment (ART) to approximately 19 million people with HIV (PWH) in the African region has prevented millions of HIV-related deaths and has resulted in rising life expectancy trends in many countries severely affected by HIV [1–3]. In addition to significant health benefits for PWH, suppressive ART also prevents the onward transmission of HIV [4,5]. In accordance with the updated 95–95–95 targets set by the Joint United Nations Program on HIV/AIDS (UNAIDS), 95% of PWH on ART should have achieved suppression of the HIV-RNA load (viral load) by 2025 [6].

Viral load monitoring during ART in low and middle-income countries (LMIC) is typically performed yearly as recommended by the WHO guidelines [7,8]. In case of viral rebound above 1000 copies/ml during ART, adherence interventions are recommended, followed by a repeat viral load within 3 months, which may confirm virological failure or demonstrate resuppression. Observational data from LMICs suggest that this practice results in prolonged continuation of a failing regimen in the face of drug resistance, leading to significant accumulation of drug resistance mutations and loss of options for follow-up therapeutic regimens [9–11]. We and others have shown that in large cohorts of PWH in routine clinical care in sub-Saharan Africa, individuals who develop viral rebound are likely to remain viremic for a prolonged period of time, and are only switched to a new active regimen after more than 1 year of viraemia [11–13]. More frequent viral load testing may lead to earlier detection of viraemia, potentially preventing accumulation of drug resistance and reducing the risk of onward HIV transmission. However, prospective data on the optimal frequency of viral load measurement are lacking.

In case virological failure is confirmed, clinical guidelines in most LMICs recommend empiric switching to a second-line treatment regimen due to the unavailability of routine drug resistance testing. This practice risks unnecessary and potentially harmful switches to second-line treatment in individuals who do not harbor drug-resistant HIV, and in whom failure is solely due to underlying nonadherence. Recently developed methods for qualitative measurement of key antiviral compounds may enable low-cost drug exposure testing at the point of care [14–16]. In retrospective studies of PWH with failure of ritonavir-boosted lopinavir (LPV/r) second-line ART, LPV exposure testing demonstrated undetectable LPV levels in roughly half of cases, indicating significant nonadherence [17,18]. Individuals with undetectable drug levels were highly unlikely to harbor drug-resistant HIV, showing that drug exposure testing could be used to guide adherence interventions and prevent unnecessary switching to second-line ART.

We performed an open-label randomized controlled trial (RCT) to assess whether increased frequency three-monthly viral load monitoring, and a strategy of step-up point-of-care drug exposure testing and drug resistance testing in case of viral rebound, can reduce time to detection and duration of viraemia and avoid unnecessary switching to second-line ART in adult PWH.

## Materials and methods

### Design and patient population

This open-label RCT was conducted at Ndlovu Medical Centre, a large treatment facility providing HIV treatment and care in Elandsdoorn, Limpopo, South Africa. Enrolment occurred between June 28, 2015, and August 31, 2017. Study procedures were integrated in clinical practice and performed by routine clinical staff in a pragmatic fashion. The study protocol (Supplementary 7) was registered at ClinicalTrials.gov (NCT03357588), and result reporting was aligned with CONSORT guidelines (Supplementary 8).

Individuals aged 18 years or above were eligible if they were either initiating or already receiving first-line ART containing either efavirenz (EFV) or nevirapine (NVP) and two nucleos(t)ide reverse transcriptase inhibitors (NRTIs). Individuals already receiving ART were only eligible if they were on ART for at least 12 months and had a viral load of less than 1000 copies/ml performed 6 months or less before enrolment. Individuals were excluded in case of any unstable medical condition at enrolment.

### Study procedures

#### Randomization

**Fig. 1:**
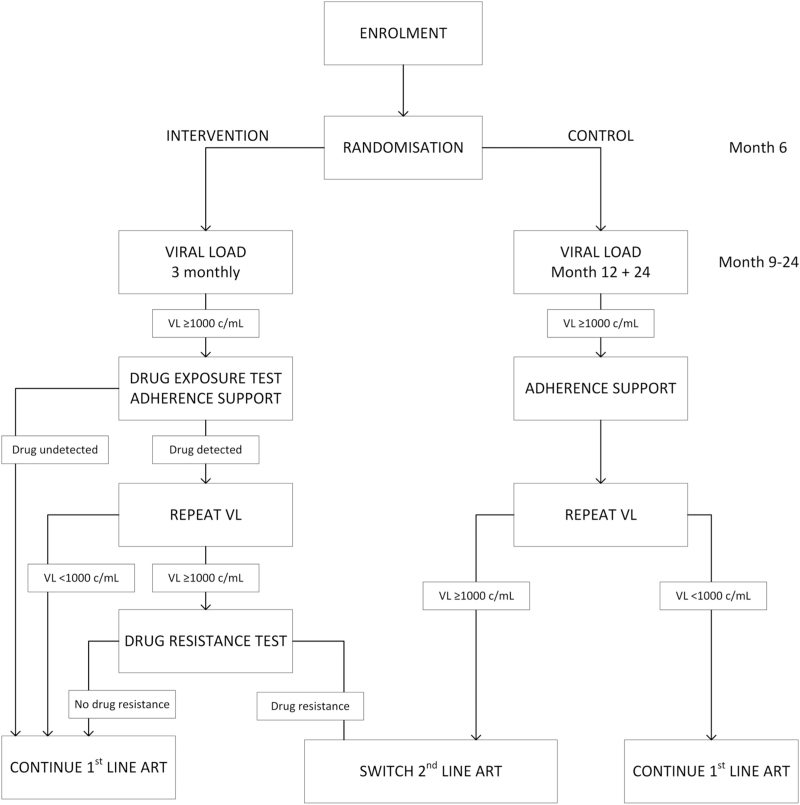
Trial design.

Randomization occurred 6 months after the start of ART in newly initiated participants and 6 months after the last annual viral load in participants on first-line ART. Newly initiating participants were only eligible for randomization if their viral load at month 6 of ART was less than 1000 copies/ml. Randomization was performed by the investigators using a computer-generated list with a 1 : 1 allocation ratio. The randomization result was reported to participants and clinicians. Study visits were performed every 3 months for a total follow-up duration of 24 months after initiation of ART and 18 months after randomization (Fig. 1).

VL, viral load.

#### Control arm procedures

Viral load measurements were performed at month 6 and 18 after randomization in the control arm, corresponding to month 12 and 24 after initiation of ART or last annual viral load measurement, in concordance with the annual viral load monitoring recommendation by the WHO [7]. In case of a viral load result above 1000 copies/ml, participants were called back after 1 month for adherence counseling and repeat viral load measurement 2 months after the initial viral load measurement. If the repeat viral load result was above 1000 copies/ml, a switch to second-line therapy consisting of ritonavir-boosted lopinavir and two NRTIs was made empirically in accordance with South African guidelines [19]. If the confirmatory viral load was below 1000 copies/ml after adherence counseling, first-line ART was maintained (Fig. 1).

#### Intervention arm procedures

Viral load monitoring was performed at every 3-month study visit after randomization in the intervention arm. In case of a viral load above 1000 copies/ml, participants were called back after 1 month and the step-up strategy was initiated. At the first return visit, adherence counseling and point-of-care drug exposure testing for EFV or NVP was performed. If drug level was detectable, viral load testing was repeated at the same visit. If the repeat viral load was above 1000 copies/ml, drug resistance testing was performed. In case of confirmed failure and drug resistance, a switch to second-line therapy consisting of ritonavir-boosted lopinavir and two NRTIs, guided by resistance testing results, was made at the second return visit. If drug exposure testing was undetectable, if the confirmatory viral load was below 1000 copies/ml, or if drug resistance testing did not detect resistance, first-line ART was maintained (Fig. 1).

#### Laboratory procedures

Drug resistance testing was performed in real-time when indicated as part of the step-up strategy in the intervention arm, and retrospectively in cases of confirmed virological failure in the control arm. Drug resistance testing and viral load testing were performed prior to start of ART in all participants newly initiating ART in the trial. Viral load testing was also performed retrospectively on all timepoints prior to detection of failure in participants with failure in the control arm. Drug exposure testing was performed as a point-of-care test using a locally validated investigational-use only immunoassay [16]. Laboratory procedures are described in detail in the supplementary materials. (Supplementary 1)

#### Adherence data

Data on self-reported adherence were collected using the CASE adherence index score at every three-monthly study visit [20]. A pill count was also performed at every three-monthly study visit.

#### Study outcomes

The primary outcomes were presence of drug resistance, accumulation of drug resistance, and unnecessary switches to second-line ART. Accumulation of drug resistance was defined as the absolute number of drug resistance mutations present, as well as the selection of the K65R mutation specifically. Unnecessary switches to second-line ART were defined as a switch in the control arm in absence of drug resistance to the first-line ART regimen as indicated by retrospective drug resistance testing.

Secondary virological outcomes were viral rebound (viral load ≥1000 copies/ml), confirmed virological failure, time to detection of rebound, and duration of viremia. Time to detection of rebound was defined as the period from start of ART for newly initiating participants or last viral load below 1000 copies/ml for participants already on ART until first detection of a viral load above 1000 copies/ml. Only viral load results that were performed in real time in the trial were considered for this outcome. Duration of viraemia was defined as the time from onset until resolution of viral load above 1000 copies/ml as determined by 12-weekly viral load testing, which was performed in real time in the intervention arm and retrospectively in the control arm.

Clinical outcomes of exploratory interest were CD4^+^ cell count change between enrolment and week 96, quality of life (QoL) as measured using the WHO QOL Bref questionnaire [21], patient mortality, and loss to follow-up. Loss to follow-up was defined as having not attended at least two consecutive 12-weekly study visits without any record of treatment collection in the interim.

#### Statistical analysis

Primary outcome analysis was performed using a modified intention-to-treat approach were every participant with at least one viral load result after randomization was included. In univariate comparisons between arms the χ^2^-test and Fisher exact tests were used for dichotomous covariables and the Student's *t*-test and Mann--Whitney *U* test for continuous covariables. For analyses of event data, the Cox proportional hazards model was used, and hazard ratios were reported along with 95% confidence intervals. The power analysis for the study is available in the supplementary materials (Supplementary 2)

#### Ethics statement

The ITREMA RCT received ethical approval from the University of Pretoria Faculty of Health Sciences Research Ethics Committee (Ref 69/2015) and the Provincial Department of Health of Limpopo, South Africa (Ref 4/2/2). All participants provided written informed consent. This study was performed in accordance with South African GCP Guidelines and principles of the Declaration of Helsinki.

## Results

### Participant characteristics

Five hundred and one participants were included, of whom 351 (70.1%) were women, with a median age of 42.4 [interquartile range (IQR) 35.6–49.2] years. 41.3% (207/501) of participants were enrolled during initiation of first-line ART. Newly initiating participants had a median CD4^+^ T-lymphocyte count of 191 [IQR 70–355] cells/μl at the start of ART. 58.7% (294/501) of participants were enrolled while already on first-line ART and within 6 months of their last viral load result. Participants already on first-line ART had a median CD4^+^ T-lymphocyte count (CD4^+^ cell count) of 142 [IQR 49–225] cells/μl at initiation of first-line ART and 523 [IQR 374–709] cells/μl at enrolment, and had received ART for a median 6.2 [4.2–8.4] years prior to enrolment. Prescribed ART consisted of TDF/FTC/EFV in 95.8% (480/501) of participants (Table 1, Supplementary 3).

**Table 1:** Characteristics of participants.

Characteristics	Units	Overall	Randomized	Control	Intervention	*P*
*n*		501	416	208	208	
Sex	female (%)	351 (70.1)	298 (71.6)	150 (72.1)	148 (71.2)	0.913
Age	median years [IQR]	42.4 [35.6–49.2]	42.9 [36.5–49.9]	44.0 [36.3–50.0]	42.8 [37.3–49.8]	0.769
CD4^+^ cell count at enrolment^a^	median cells/μl [IQR]	376 [191–582]	398 [217–622]	403 [230–651]	393 [203–575]	0.469
CD4^+^ cell count at start ART (pretreated group)	median cells/μl [IQR]	142 [49–225]	139 [47–224]	147 [50–226]	124 [41–214]	0.339
Time on ART (pretreated group)	median days [IQR]	2243 [1519–3048]	2249 [1520–3050]	2246 [1522–3074]	2262 [1519–3044]	0.801
Most recent VL (pretreated group)						0.423
	<50 copies/ml	135 (47.4)	124 (46.6)	54 (42.2)	70 (50.7)	
	50–200 copies/ml	65 (22.8)	63 (23.7)	31 (24.2)	32 (23.2)	
	200–400 copies/ml	27 (9.5)	25 (9.4)	15 (11.7)	10 (7.2)	
	400–999 copies/ml	56 (19.6)	53 (19.9)	28 (21.9)	25 (18.1)	
	≥1000 copies/ml	2 (0.7)	1 (0.4)	0 (0.0)	1 (0.7)	
VL at enrolment (newly initiated group)						0.880
	<50 copies/ml	7 (3.4)	5 (3.6)	3 (4.1)	2 (3.0)	
	50–200 copies/ml	10 (4.9)	7 (5.0)	4 (5.4)	3 (4.5)	
	200–400 copies/ml	20 (9.8)	14 (10.0)	9 (12.2)	5 (7.6)	
	400–999 copies/ml	75 (36.6)	49 (35.0)	24 (32.4)	25 (37.9)	
	≥1000 copies/ml	93 (45.4)	65 (46.4)	34 (45.9)	31 (47.0)	
Regimen						NA
	TDF/FTC/EFV	480 (95.8)	396 (95.2)	198 (95.2)	198 (95.2)	
	ABC/3TC/EFV	13 (2.6)	13 (3.1)	7 (3.4)	6 (2.9)	
	TDF/FTC/NVP	4 (0.8)	4 (1.0)	3 (1.4)	1 (0.5)	
	ABC/3TC/NVP	2 (0.4)	2 (0.5)	0 (0.0)	2 (1.0)	
	ZDV/3TC/EFV	1 (0.2)	1 (0.2)	0 (0.0)	1 (0.5)	
	ABC/3TC/LPVr^b^	1 (0.2)	0 (0.0)	0 (0.0)	0 (0.0)	
WHO stage						0.707
	I	411 (82.0)	361 (86.8)	178 (85.6)	183 (88.0)	
	II	45 (9.0)	28 (6.7)	17 (8.2)	11 (5.3)	
	III	38 (7.6)	25 (6.0)	12 (5.8)	13 (6.2)	
	IV	7 (1.4)	2 (0.5)	1 (0.5)	1 (0.5)	
Karnofsky performance score	mean (sd)	91.74 (4.92)	92.13 (4.71)	91.81 (4.97)	92.45 (4.42)	0.169
Education						0.180
	primary	122 (24.4)	105 (25.2)	59 (28.4)	46 (22.1)	
	secondary	348 (69.5)	282 (67.8)	138 (66.3)	144 (69.2)	
	tertiary	31 (6.2)	29 (7.0)	11 (5.3)	18 (8.7)	
Prior ART	Prior ART exposure (%)	38 (7.6)	31 (7.5)	14 (6.7)	17 (8.2)	0.709
Treatment status at trial entry	newly initiated (%)	207 (41.3)	141 (33.9)	73 (35.1)	68 (32.7)	0.679

Results displayed as mean (standard deviation), median [interquartile range], or count (percentage). 3TC, lamivudine; ABC, abacavir; ART, antiretroviral treatment; c/mL, copies/milliliter; CD4, CD4^+^ T-lymphocyte count; EFV, efavirenz; FTC, emtricitabine; IQR, interquartile range; LPVr, ritonavir-boosted lopinavir; NVP, nevirapine; TDF, tenofovir disoproxil fumarate; VL, HIV-RNA viral load; ZDV, zidovudine.

a CD4^+^ cell count at the start of ART for individuals newly initiated on ART is the same as CD4^+^ cell count at enrolment.

b One individual receiving LPVr as first-line ART was enrolled but subsequently excluded.

#### Randomization and follow-up

**Fig. 2:**
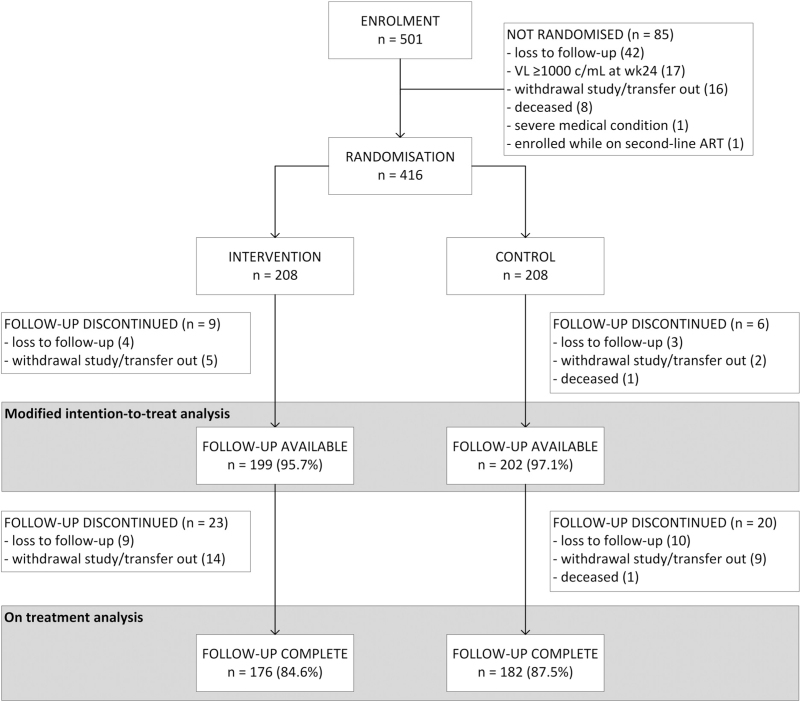
CONSORT flow diagram.

VL, viral load; wk, week.

Four hundred and sixteen participants met criteria for randomization after study week 24 and 208 participants were randomized to each arm. There were no statistically significant differences in the distribution of key clinical and demographic variables between the control and intervention arms (Table 1). Of randomized patients, any virological follow-up was available in 96.4% (401/416) and these participants were entered into modified intention-to-treat analysis. Full follow-up was available in 86.1% (358/416) of participants (Fig. 2, Supplementary 4).

#### Participants excluded from randomization

Main reasons for nonrandomization were loss to follow-up in 49.4% (42/85) and having a viral load above 1000 copies/ml at month 6 in 20.0% (17/85) of participants (Fig. 2). Participants who did not meet criteria for randomization were more likely to have newly initiated ART, had a lower median CD4^+^ cell count and lower median performance score, were at a more advanced WHO stage, and were younger on average (*P* < 0.001 for all comparisons) (Supplementary 4).

#### Viral rebound

Frequency of viral rebound was 9.0% (18/199) in the intervention arm versus 11.9% (24/202) in the control arm [odds ratio (OR) = 0.74; 95% CI 0.36–1.47, *P* = 0.445]. Time to detection of viral rebound was 375 days [IQR: 348–515] in the intervention and 360 days [IQR: 338–464] in the control arm (*P* = 0.683). After rebound, the majority of participants in both arms achieved resuppression (viral load <1000 copies/ml) on the first-line ART regimen, 82.4% (14/17) in the intervention arm versus 78.9% (15/19) in the control arm (OR 1.24; 95% CI 0.17–10.00, *P* = 1.00) (Table 2). In the intervention arm, drug exposure testing after viral rebound was positive in 92.3% (12/13).

**Table 2:** Primary outcome analysis.

*Modified intention-to-treat (mITT) analysis*				
	intervention arm (*n* = 199)	Control arm (*n* = 202)	OR/HR [95% CI]	*P*
Viral rebound	9.0% (18)	11.9% (24)	OR: 0.74 [0.36–1.47]	0.445
Switch to second-line ART^a^	1.0% (2)	1.5% (3)	OR: 0.67 [0.05–6.68]	1
Time to viral rebound	375 days [IQR: 348–515]	360 days [IQR: 338–464]	HR: 0.88 [0.46–1.66]	0.683

*On-treatment analysis*				
	Intervention arm (*n* = 176)	Control arm (*n* = 182)	OR/HR [95% CI]	*P*
Viral rebound	9.7% (17)	12.1% (22)	OR: 0.78 [0.37–1.60]	0.570
Switch to second-line ART^a^	1.1% (2)	1.6% (3)	OR: 0.71 [0.05–7.21]	1
Time to viral rebound	365 days [IQR: 348–446]	356 days [IQR: 338–533]	HR: 0.90 [0.47–1.72]	0.742

*Patients with viral rebound (mITT)*				
	Intervention arm (*n* = 18)	Control arm (*n* = 24)	OR [95%CI]	*P*
Duration of viral rebound^b^	87 days [IQR: 70–110]	101 days [IQR: 78–213]	NA	0.423
Resuppression on first-line ART^a^	82.4% (14/17)	78.9% (15/19)	OR: 1.24 [0.17–10.00]	1

Proportions were analyzed using chi-squared tests and time-to-event data were analyzed using Cox proportional hazard models. 95% CI, 95% confidence interval; HR, hazard ratio; IQR, interquartile range; OR, odds ratio.

a Fisher's Exact test was used due to low expected event counts.

b Wilcoxon rank sum test was used, and each instance of viral rebound was assessed separately (participant 311 contributed two rebound episodes).

#### Confirmed virological failure, drug resistance, and switch

Confirmed virological failure occurred in 1.5% (3/199) of cases in the intervention arm versus 1.5% (3/202) of cases in the control arm (OR = 1.02; 95% CI 0.13–7.67, *P* = 1.00). In the intervention arm, only one participant had a positive drug level and detected drug resistance and was switched. Switch to second-line ART was prevented in the remaining two participants by negative drug level in one case and absence of detectable drug resistance in the other case. The participant without detectable drug resistance developed a second episode of failure on first-line ART. Drug resistance was then detected, and the participant was eventually switched. In the control arm, all three participants were switched to second-line ART as per the standard clinical protocol. Statistical analysis for drug resistance selection and accumulation outcomes were not performed due to low case numbers (Table 3).

**Table 3:** Drug resistance in participants with confirmed virological failure.

	At viral rebound					At start of treatment	
Participant	Time on ART	Viral load	Drug exposure testing	NRTI resistance	NNRTI resistance	Viral load	Drug resistance
*Intervention*							
ITREMA 078	86.6 weeks	230 445	negative	-^b^	-^b^	197 527	None
ITREMA 290	322 weeks	49 249	positive	-	K103KN, V106VM	NA^c^	NA^c^
ITREMA 311^a^	259.9 weeks	1190	positive	-	-	NA^c^	NA^c^
ITREMA 311^a^	283.4 weeks	1921	positive	K65R, M184V	K103N, V106M	NA^c^	NA^c^
*Control*							
ITREMA 039	48.1 weeks	1380	not done	M184MI^b^	M230I^b^	218 115	None
ITREMA 094	50.4 weeks	79 100	not done	M184V^b^	K103N, V106VM^b^	111 916	None
ITREMA 454	477.6 weeks	992 000	not done	-^b^	-^b^	NA^c^	NA^c^

a Participant 311 had two separate episodes of failure, and the intervention strategy, including drug resistance testing, was therefore performed twice.

b resistance testing performed retrospectively. Viral load, quantitative HIV-RNA, displayed in copies/ml.

c pretreatment viral load and drug resistance testing not done as participant was enrolled while already on ART at enrolment.

#### Duration of viraemia and time on failing regimen

In participants with viral rebound and available follow-up (*n* = 42), the median duration of viraemia was 87 days [IQR 70–110] in the intervention group and 101 days [IQR 78–213] in the control group (*P* = 0.423) (Table 2). In participants with confirmed virological failure who were switched to second-line ART (*n* = 5), the median time spent on a failing regimen was 196 days [IQR 173–219] in the intervention group versus 295 days [IQR 225–308] in the control group (*P* = 0.4).

#### Adherence

Markers of adherence did not differ between groups. The median value of the lowest pill count score and lowest self-reported adherence score obtained during the 96 weeks follow-up duration was similar between arms (*P* = 0.325 for pill count and *P* = 0.641 for self-reported adherence) (Supplementary 5).

#### Immunological failure, morbidity, and mortality

CD4^+^ cell count recovery between enrolment and week 96 was 69 [IQR −36 to 182] cells/μl in the intervention arm versus 45 [IQR −44 to 170] cells/μl in the control arm (*P* = 0.503). Mortality during follow-up was 2.0% (10/501). In the intention-to-treat population, no deaths occurred in the intervention arm versus one death in the control arm (OR 0.00; 95% CI 0.00–39.60, *P* = 1.000) (Supplementary 5).

#### Quality of life

Participant-reported quality of life (QOL) revealed a slight downward trend over time in the study. Change in QOL between enrolment and week 96 was similar between arms [−4.3% (IQR −12.9 to 4.3) in control versus −4.3% (IQR −12.9 to 2.2) in intervention arm; *P* = 0.692] (Supplementary 5).

#### Follow-up of unrandomized participants

Eighty-one participants were not randomized. Any follow-up data were available for 18 participants and full 96 weeks follow-up data were available for 14 participants (Supplementary 6). Of participants with follow-up, 92.9% (13/14) had a viral load above 1000 copies/ml at their first viral load measurement in the trial. All participants went on to develop confirmed virological failure and were switched to second-line ART during trial follow-up. 61.5% (8/13) of participants had a viral load below 1000 copies/ml at week 96 on second-line ART.

## Discussion

Clinical guidelines conflict in their recommendations on the frequency of viral load monitoring. To our knowledge, this study is the first to prospectively compare different frequencies of viral load measurement. It showed no statistically significant effect of more frequent testing on time to detection of viral rebound and duration of viraemia. Although a larger sample size may have shown a statistically significant effect, the clinical impact of this intervention is likely very limited. Extrapolating from the results encountered in this study, approximately 40 additional viral loads would need to be performed to achieve a 15-day shortening of the duration of one viremic episode, indicating a high cost for a modest reduction in duration. The results of this study provide evidence in favor of maintaining the standard of care of annual viral load monitoring in LMIC.

The results highlight the outsized importance of the first viral load test after initiation of ART compared with subsequent testing and illustrates the prognostic importance of achieving initial virological response to ART. Only six randomized participants developed confirmed virological failure during follow-up. In contrast, 13 cases of confirmed virological failure occurred at the initial 6-month viral load test prior to randomization and in most cases concerned participants newly initiating ART. These results argue for close follow-up of the initial viral load measurement, and urgent clinical intervention if initial suppression is not achieved.

Healthcare worker response to viraemia in LMIC is often delayed, leading to prolonged duration of viremic episodes prior to clinical intervention [11–13]. Our intensified monitoring strategy sought to address this by initiating step-up point-of-care drug exposure testing and drug resistance testing after viral rebound. This strategy was successfully implemented, demonstrating its potential ability to avoid unnecessary switches to second-line ART and to reduce the duration of viraemia. However, its efficacy in improving these outcomes could not be formally assessed due to low rates of confirmed virological failure in this trial.

The low rate of confirmed failure was a result of the high rate of resuppression at the confirmatory viral load test in most participants who developed viral rebound in the study, which exceeded resuppression rates reported in other cohorts [22]. This suggests frequent occurrence of intermittent episodes of nonadherence in our participants. Surprisingly, drug exposure testing after rebound was positive in over 90% of cases, rendering the yield of this test for detection of such nonadherence almost negligibly low. This could be due to the implementation of the test at the clinical visit after detection of viral rebound, at which point resuppression was often already achieved, and may additionally be attributable to the long half-life of EFV, which commonly remains detectable in plasma between several days to weeks after treatment cessation, significantly longer than other components of ART [16].

Drug resistance testing revealed NNRTI resistance in two-thirds of patients with confirmed virological failure in this study, which is in line with surveys from LMIC of patients on NNRTI-based first-line ART [23]. The high prevalence of drug resistance leaves limited potential for drug resistance testing as a means of avoiding unnecessary switches to second-line ART. Confirming this, a recent trial evaluating the clinical value of drug resistance testing alone in patients with viral rebound of NNRTI-based ART showed no benefit of implementation of drug resistance testing, in part due to the high pretest probability of NNRTI drug resistance in patients with rebound [24].

Although these results confirm the utility of current WHO recommendations for empiric switching after confirmed failure of NNRTI-based ART, the global transition to first-line ART with the integrase strand-transfer inhibitor (InSTI) dolutegravir (DTG) requires a rethink of conventional ART monitoring strategies. The high genetic barrier to drug resistance of DTG potentially increases the benefit of drug resistance testing, as the likelihood of treatment-emergent InSTI-resistance after failure of first-line treatment with DTG is very low [25,26]. Moreover, empiric switching may potentially be harmful, as DTG has shown superiority in head-to-head comparisons with ritonavir-boosted lopinavir in second-line ART [27]. On the contrary, failure in the presence of initial DTG-based ART is most likely due to nonadherence in the absence of resistance, and most patients with viral rebound on these regimens achieve viral resuppression [28].

Sustainable and cost-effective strategies to detect InSTI resistance in LMIC are needed for individual patient care as well as for surveillance of InSTI resistance on the population level. The development of such strategies is therefore an urgent research priority. Drug exposure testing is an effective screening tool for drug resistance when used during virological failure of regimens with high genetic barriers to resistance such as ritonavir-boosted lopinavir [17,18]. It could be expected that drug exposure testing may yield similar results as a screening test for InSTI resistance during treatment with DTG, given its high genetic barrier to resistance. This hypothesis is currently being tested in one RCT, which evaluates an intensified monitoring intervention similar to the strategy described in this trial, combined with point-of-care viral load monitoring, in patients on DTG-based ART [29].

### Limitations

This study attempted to assess the implementation and clinical value of frequent viral load monitoring combined with a step-up strategy of point-of-care drug exposure testing and drug resistance testing. Although the study was implemented in a pragmatic fashion to ensure close correlation with routine care in South Africa, the single-center nature of the study may have limited the generalizability of results. Although the required total sample size was attained, lower than expected rates of confirmed virological failure resulted in a loss of statistical power to demonstrate the efficacy of the proposed study intervention. Lastly, this study was performed prior to the transition of NNRTI-based to DTG-based first-line ART in South Africa, thus limiting the ability to directly translate the study results to the current standard of care.

### Conclusion

We report the successful implementation of an intensified ART monitoring strategy of routine three-monthly viral load testing and a step-up strategy after detection of virological failure, consisting of drug exposure testing and drug resistance testing, in a resource-limited rural clinical setting. This randomized study shows that three-monthly viral load testing did not significantly shorten the duration of viremia compared with standard-of-care annual viral load testing, providing randomized trial data to support the annual viral load monitoring interval maintained by the WHO. The efficacy of the step-up strategy could not be statistically evaluated owing to high rates of treatment success in the study.

## Acknowledgements

The authors thank the ITREMA participants for their participation, and the staff at Ndlovu Medical Centre for their dedication in the implementation of the ITREMA trial. The authors acknowledge the HIV geno team at UMC Utrecht for assistance in genotypic resistance testing. The authors thank Marloes Frijters, Maartje van der Molen, Stijn Talboom, Sena Ebbers, and Natasja van der Meer for assistance with data capturing, Josien Straver for assistance with project management, and Emma-Lena Maris and Liset de Vries for assistance with dissemination and training activities for the project.

The authors would like to acknowledge the members of the advisory board for the ITREMA trial: Andy Gray, Osama Hamoudah, Elliot Raizes, Douglas Richman, Jonathan Schapiro, and Annelies van der Vorm.

A.W., R.S., M.N., H.T., F.V., and L.H. designed the study, drafted the study protocol, and obtained funding. R.t.H. and D.B. provided expertise and support for drug exposure testing. D.d.J., L.H., R.S., M.N., and A.W. provided drug resistance testing and interpretation of drug resistance results for the study. L.H., H.T., and W.D. performed or oversaw clinical duties and operational logistics for the study. L.H. and S.V. developed the adherence counseling procedures that were used in the study. L.H. drafted the first version of the manuscript. All authors read and approved the submitted version of the manuscript.

This project was supported by the Netherlands Organisation for Health Research and Development (ZonMW) and NWO-WOTRO Science for Global Development through grant no. 205300004 and additional funding for dissemination and implementation (VIMP). ARK Diagnostics, Inc. provided immunoassay drug exposure testing kits free of charge, but was not involved in the design, execution, or analysis of the study.

### Conflicts of interest

The authors report no conflicts of interest related to this study or its results.

## Supplementary Material

Supplemental Digital Content

## Supplementary Material

Supplemental Digital Content
