# Supplementary material for: A randomized study of intensified antiretroviral treatment monitoring versus standard-of-care for prevention of drug resistance and antiretroviral treatment switch
Source: AIDS. 2022 Oct 4;36(14):1959–68. doi: 10.1097/QAD.0000000000003349 (PMC9612712; doi:10.1097/QAD.0000000000003349)
Supplement: Supplemental Digital Content [file aids-36-1959-s001.docx]

**A Randomised Study of an Intensified Antiretroviral Treatment Monitoring Strategy Versus Standard-Of-Care for the Prevention of HIV Drug Resistance Accumulation and Treatment Switch in Resource-Limited Settings: Week 96 Results of the ITREMA Trial**

Supplementary materials

Contents

**Supplementary 1** – Laboratory procedures

**Supplementary 2** – Sample size calculation

**Supplementary 3** – Table of newly initiated participants versus participants already on ART

**Supplementary 4** – Table of randomised versus unrandomised participants

**Supplementary 5** – Virological suppression rates in trial participants

**Supplementary 6** – Secondary study outcomes

**Supplementary 7** – Outcomes of unrandomised participants

**Supplementary 8** – Study protocol (separate document)

**Supplementary 9** – CONSORT checklist (separate document)

**Supplementary 1**

Viral load testing: Quantitative measurement of HIV-1 RNA was performed on plasma collected in plasma preparation tube (PPT) vacutainers using the Roche COBAS^®^ AmpliPrep/COBAS^®^ TaqMan^®^ HIV-1 Test, version 2.0 (Roche Molecular Systems, USA). Results above 50 copies/mL were reported quantitatively, and results below this threshold were reported as “lower than detectable limit”. Batchwise retrospective analyses were performed on stored plasma samples of participants at ART initiation, and in participants in the control arm with therapy failure on all available study samples to determine duration of viraemia prior to detection. Testing was performed by two laboratory providers, the South African National Health Laboratory Service (Johannesburg, Gauteng, RSA) and Toga laboratories (Pty) Ltd (Johannesburg, Gauteng, RSA). Both laboratories are accredited medical pathology laboratories by the South African National Accreditation System (SANAS).

Drug exposure testing: Drug exposure testing was performed in case of a VL ≥1000 copies/ml in the intervention arm. Drug exposure testing was performed at the first return visit, one month after the visit at which rebound was detected, using EDTA-derived plasma obtained at the call back visit. Drug exposure testing was performed on site using a validated immunoassay that was implemented at Ndlovu Medical Centre as described previously.^1^ Briefly, immunoassays targeting EFV and NVP supplied by ARK Diagnostics, Inc. (Fremont, CA, USA) were implemented on a benchtop chemistry analyser (Indiko Plus chemistry system, Thermo-Scientific, Waltham, MA, USA) at Ndlovu Medical Centre. Test results were generated while the participant was waiting and were reported in a qualitative fashion as either “detectable drug level” or “no detectable drug level”, the latter indicating at least several days of non-adherence over the last week.^1^ This result was reported back to the clinician, who informed the participant of the result during the same clinic visit.

Drug resistance testing: Genotypic drug resistance testing was performed on dried blood spots (DBS) in the intervention arm in case of a confirmed VL of ≥1000 copies/ml and a detectable drug level. Drug resistance testing was additionally performed retrospectively in cases of failure in the control arm and prior to start of ART in participants newly initiating ART in the trial. EDTA-derived whole blood collected at the first return visit was spotted in five spots of 50µL each on a Protein Saver 903 card (Whatman Nederland B.V., Den Bosch, The Netherlands) and left to dry overnight prior to packaging in a zip-lock bag containing desiccant. In case the confirmatory VL result was ≥1000 copies/ml, the DBS card was shipped on room temperature to a WHO reference laboratory for drug resistance testing at the University Medical Center Utrecht, Utrecht, The Netherlands. Population-based sequencing of the *reverse transcriptase* gene of HIV-1 was performed using an assay optimised for non-B HIV-1 subtypes. This assay has been validated for use on DBS. Briefly, the assay consists of a single-round one-tube RT-PCR to amplify the viral RT-gene between codons 40-238, followed by population sequencing of the amplified fragment in both directions using 2 primers only.^2,3^ In case of failure of this assay, an alternative sequencing protocol for the *protease* and *reverse transcriptase* genes was performed.^4^

References:

1. Hermans LE, Nijhuis M, Tempelman HA, et al. Point-of-Care Detection of Nonadherence to Antiretroviral Treatment for HIV-1 in Resource-Limited Settings Using Drug Level Testing for Efavirenz, Lopinavir, and Dolutegravir: A Validation and Pharmacokinetic Simulation Study. *J Acquir Immune Defic Syndr*. 2021;87(4):1072-1078. doi:10.1097/QAI.0000000000002681

2. Aitken S, Slabbert M, Schrooders P, Tempelman H, Schuurman R, Wensing A. HIV-1 resistance testing on dried blood spots enables individual patient management in arural South-African setting. In: *Towards Virological Monitoring of HIV-1 Drug Resistance in Resource-Limited Settings.* ; 2013.

3. Aitken SC, Bronze M, Wallis CL, et al. A pragmatic approach to HIV-1 drug resistance determination in resource-limited settings by use of a novel genotyping assay targeting the reverse transcriptase-encoding region only. *J Clin Microbiol*. 2013;51(6):1757-1761. doi:10.1128/JCM.00118-13

4. Aitken SC, Kliphuis A, Wallis CL, et al. Development and evaluation of an assay for HIV-1 protease and reverse transcriptase drug resistance genotyping of all major group-M subtypes. *Journal of Clinical Virology*. 2012;54(1):21-25. doi:10.1016/j.jcv.2012.01.010

**Supplementary 2**

Sample size calculation: The primary sample size calculation was based on observed prevalence of the K65R mutation during first-line ART failure in LMIC (approximately 70%) versus high-income settings with stringent monitoring (approximately 17%).^5,6^ Detection of this difference would require 83 participants per arm. Given the differential selection of K65R by HIV-1 subtype C and subtype B, an additional power analysis was based on expected differences in selection of multiple resistance mutations between arms, derived from local pilot study, where an increase of 2.8 to 4.3 NNRTI and NRTI mutations per additional 6-12 months of failing therapy was observed.^7^ Detection of this difference would require 125 participants per arm. Sample size calculation was not performed for the outcome of unnecessary switches to second-line ART given the absence of available estimates for this outcome. Instead, an additional calculation for the secondary outcome of viral rebound was performed. The cumulative prevalence of viral rebound was anticipated to be 15% in the control arm versus 5% in the intervention arm. 216 participants per arm would be required to detect this difference. All sample size calculations were performed assuming a 1-*α* of 0.9 and a *β* of 0.05, and assuming an annual loss to follow-up rate and virological failure rate of 10% each.

References:

5. Sunpath H, Wu B, Gordon M. High rate of K65R for ART naïve patients with subtype C HIV infection failing a TDF-containing first-line regimen in South Africa. *AIDS,* 2012;26(13):1679-1684. doi:10.1097/QAD.0b013e328356886d.

6. Theys K, Vercauteren J, Snoeck J, et al. HIV-1 subtype is an independent predictor of reverse transcriptase mutation K65R in HIV-1 patients treated with combination antiretroviral therapy including tenofovir. *Antimicrob Agents Chemother*. 2013;57(2):1053-1056. doi:10.1128/AAC.01668-12

7. Barth RE, Aitken SC, Tempelman H, et al. Accumulation of drug resistance and loss of therapeutic options precede commonly used criteria for treatment failure in HIV-1 subtype-C-infected patients. *Antivir Ther*. 2012;17(2):377-386. doi:10.3851/IMP2010

**Supplementary 3**

Baseline table: Newly initiated participants versus participants already on ART

**Supplementary 4**

Baseline table: randomised versus unrandomised participants

**Supplementary 5**

**
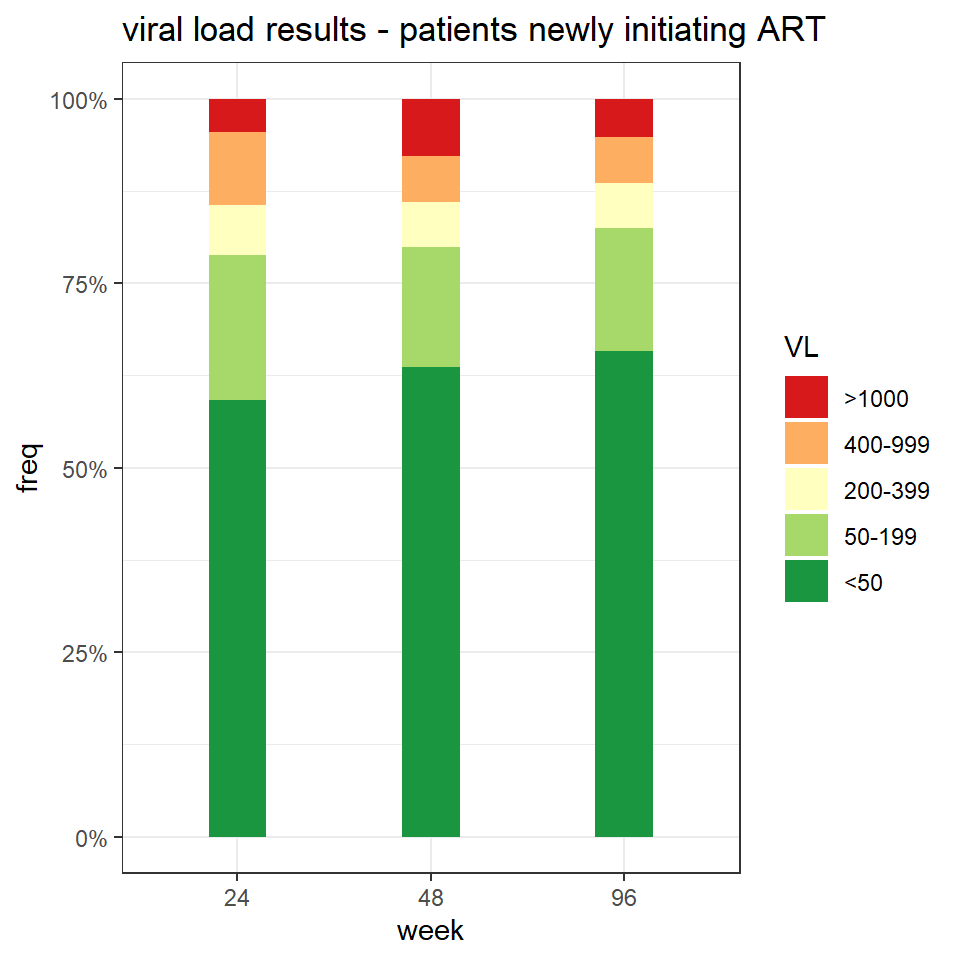

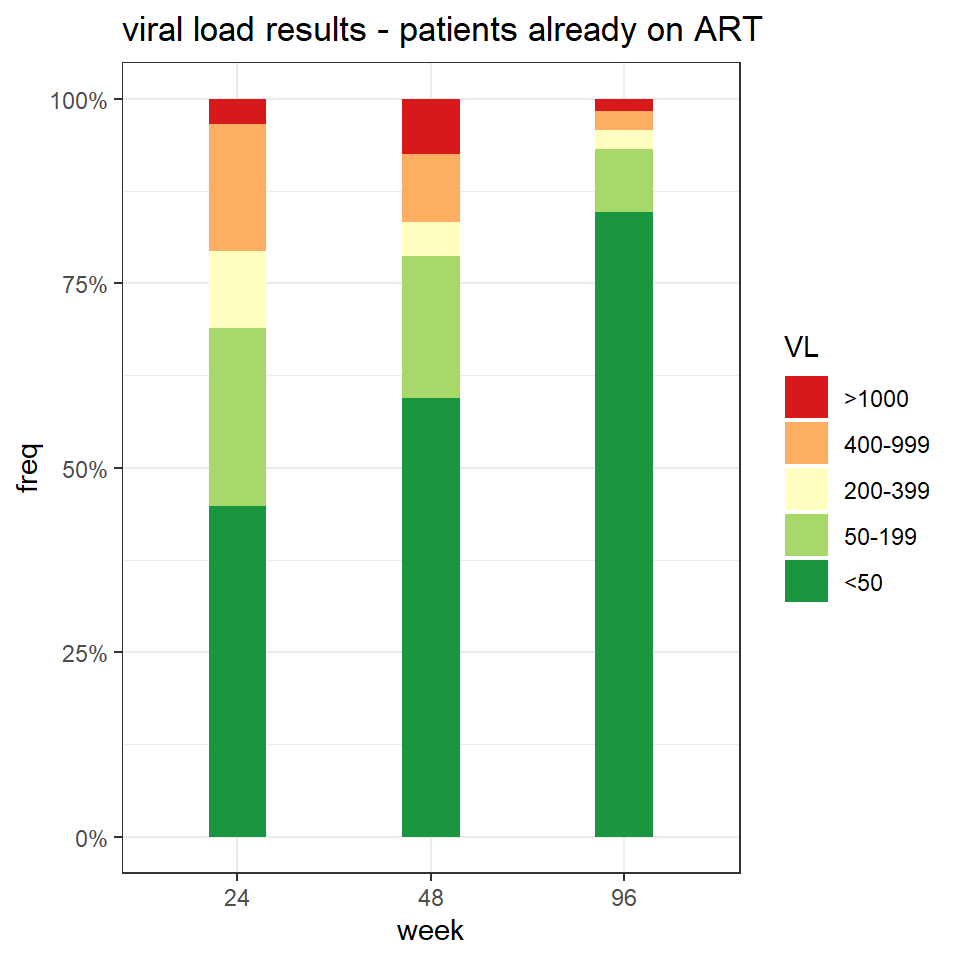
**Virological suppression rates

**Supplementary 6**

Secondary study outcomes

**Supplementary 7**

Outcomes of unrandomised participants
