# Supplementary material for: A randomized study of intensified antiretroviral treatment monitoring versus standard-of-care for prevention of drug resistance and antiretroviral treatment switch
Source: AIDS. 2022 Oct 4;36(14):1959–68. doi: 10.1097/QAD.0000000000003349 (PMC9612712; doi:10.1097/QAD.0000000000003349)
Supplement: Supplemental Digital Content [file aids-36-1959-s002.pdf]

RESEARCH PROTOCOL

EVALUATION OF AN **I**NTENSIFIED **TRE**ATMENT **M**ONITORING STRATEGY TO PREVENT **A**CCUMULATION AND SPREAD OF HIV-1 DRUG RESISTANCE IN LOW- AND MIDDLE-INCOME COUNTRIES; THE **ITREMA** PROJECT.

ITREMA

Confidential

Version 6.0

Date: August 22nd, 2016

EVALUATION OF AN INTENSIFIED TREATMENT MONITORING STRATEGY TO PREVENT ACCUMULATION AND SPREAD OF HIV-1 DRUG RESISTANCE IN LOW- AND MIDDLE-INCOME COUNTRIES; THE ITREMA PROJECT.

**Protocol ID**	69/2015
**Short title**	ITREMA
**Version**	Version 6.0
**Date**	August 22nd, 2016
**Coordinating investigator**	**Annemarie Wensing, MD PhD** Virology, Department of Medical Microbiology, University Medical Center Utrecht, Utrecht (UMCU), The Netherlands
**Principal investigator**	**Hugo Tempelman, MD** Ndlovu Care Group, Groblersdal, Limpopo
**Co-Principal investigators**	**David Burger, PharmD, PhD** Department of Pharmacy, Radboud Medical Center (RadboudMC), Nijmegen, The Netherlands<br>**Francois Venter, MD, PhD** Wits Institute for Sexual & Reproductive Health, HIV and Related Diseases (WITS RHI), Johannesburg<br>**Mirjam Kretzschmar, PhD** Dynamics of Infectious Diseases, UMCU<br>**Trudie Knijn, PhD** Interdisciplinary Social Science, Utrecht University, The Netherlands
**Co-investigators**	**Rob Schuurman, PhD** Virology, Department of Medical Microbiology, UMCU<br>**Monique Nijhuis, PhD** Virology, Department of Medical Microbiology, UMCU<br>**Mariette Slabbert, MBA** WITS RHI<br>**Rob ter Heine, PharmD, PhD**, Department of Pharmacy, RadboudMC, Nijmegen, The Netherlands<br>**Sigrid Vervoort, PhD** Department of Internal Medicine & Infectious diseases, UMCU<br>**Kiki Tesselaar, PhD** Department of Immunology, UMCU
**Coordinating junior researcher**	**Lucas Hermans, MD** Virology, Department of Medical Microbiology, UMCU
**Collaborators NRC**	**Roel Coutinho, MD, PhD** Department of Epidemiology, UMCU<br>**Walter Devillé, MD, PhD** Department of Epidemiology, UMCU<br>**Susan Aitken, PhD** Ndlovu Care Group, Groblersdal, Limpopo
**Sponsor**	**The Ndlovu Research Consortium (NRC)** consisting of the UMCU, Utrecht University, Utrecht (NL), the University of The Witwatersrand, Johannesburg (SA) and Ndlovu Care Group, Groblersdal (SA).
**Subsidising parties**	**ZonMW/WOTRO** (The Netherlands Organisation for Health Research and Development in conjunction with WOTRO Science for Global Development), grant No. 205300004
**Study Site**	Ndlovu Medical Centre, Elandsdoorn, Limpopo, South Africa
**Laboratory sites**	On-site laboratory: Toga laboratories, location Ndlovu, Elandsdoorn, Limpopo, South Africa<br>WHO Reference Laboratory for HIV drug resistance, UMCU, The Netherlands<br>Laboratory of Immunology, UMCU, The Netherlands<br>Laboratory of Pharmacy, Radboud Medical Center, Nijmegen, The Netherlands

Confidential

TABLE OF CONTENTS

List of abbreviations 5

1 SUMMARY 6

2 INTRODUCTION AND RATIONALE 10
- 2.1 Introduction 10
- 2.2 Rationale 12

3 OBJECTIVES 15
- 3.1 Primary objective – Virological effects of intensified monitoring 15
- 3.2 Secondary objectives 15
  - 3.2.1 Clinical effects of intensified monitoring 15
  - 3.2.2 Feasibility of intensified monitoring 15
  - 3.2.3 Effect on transmission of HIV 15
  - 3.2.4 Cost-effectiveness of intensified monitoring 15
  - 3.2.5 Risk factors for therapy failure 15
  - 3.2.6 Virological, immunological and pharmacological determinants of therapy failure 16

4 STUDY DESIGN AND PATIENT POPULATION 17
- 4.1 Study format 17
- 4.2 Population 17
- 4.3 Inclusion criteria (study baseline) 18
- 4.4 Exclusion criteria (study baseline) 18
- 4.5 Randomization criteria (month 6) 18

5 DESIGN & METHODS 19
- 5.1 Study design 19
  - 5.1.1 Inclusion, observational follow-up & randomisation (baseline to month 6 of ART) 19
  - 5.1.2 Control arm follow-up – standard of care HIV treatment monitoring 19
  - 5.1.3 Intervention arm follow-up – Intensified HIV treatment monitoring 20
- 5.2 Methods and procedures 24
  - 5.2.1 Laboratory setting 24
  - 5.2.2 Viral load determination 24
  - 5.2.3 Clinical assessment 24
  - 5.2.4 Follow-up of elevated viral load measurement 25
  - 5.2.5 Drug Level Monitoring (DLM) 26
  - 5.2.6 Dried Blood Spot-based Drug Resistance Testing 26
  - 5.2.7 Questionnaires 26
  - 5.2.8 Determinants of therapy failure 28
- 5.3 Study visits and procedures per visit 28
  - 5.3.1 Visits 28
- 5.4 Laboratory procedures and storage of blood 31
- 5.5 Withdrawal of individual participants 32
- 5.6 Replacement of individual subjects after withdrawal 32

6 OUTCOMES AND ANALYSIS OF RESULTS 35
- 6.1 Virological and clinical effects of intensified monitoring 35
- 6.2 Feasibility of intensified monitoring 35
- 6.3 Cost-effectiveness of intensified monitoring 36
- 6.4 Effect on transmission of HIV 37

Confidential

6.5 Risk factors for therapy failure 37
6.6 Determinants of therapy failure 38
6.7 Statistical methods 38
6.7.1 Analysis of clinical study outcomes 38
6.7.2 Analysis of clinical study outcomes 38
6.7.3 Sample size calculation 38
7 ETHICAL CONSIDERATIONS 40
7.1 Regulation statement 40
7.2 Recruitment and consent 40
7.2.1 Timelines 40
7.2.2 Recruitment procedure 40
7.2.3 Informed consent 40
7.2.4 Randomisation procedure 41
7.3 Benefits and risks assessment 41
7.3.1 Potential benefits associated with participation 41
7.3.2 Potential risks associated with participation 41
7.4 Incentives 42
8 ADMINISTRATIVE ASPECTS, MONITORING AND PUBLICATION 43
8.1 Handling and storage of data and documents 43
8.1.1 Data collection 43
8.1.2 Patient confidentiality 43
8.1.3 Record retention 43
8.2 Monitoring and Quality Assurance 43
8.3 Amendments 43
8.4 Implementation and dissemination 44
8.4.1 Implementation 44
8.4.2 Dissemination 44
8.5 Interim and end of study reports 44
8.6 Public disclosure and publication policy 45
9 REFERENCES 46

Confidential

LIST OF ABBREVIATIONS

**ACTG**	AIDS Clinical Trial Group
**AIDS**	Acquired Immune Deficiency Syndrome
**ART**	combination Anti-Retroviral Treatment
**CD4**	CD4-positive T-lymphocyte count
**DBS**	Dried Blood Spot
**DLM**	Drug level monitoring
**EDTA**	Ethylenediaminetetraacetic Acid
**EFV**	Efavirenz
**GCP**	Good Clinical Practice
**HCT**	HIV Counseling and Testing
**HIV**	Human Immunodeficiency Virus
**IC**	Informed Consent
**ICER**	Incremental Cost-Effectiveness Ratio
**LMIC**	Lower and Middle Income Countries
**NCG**	Ndlovu Care Group
**NMC**	Ndlovu Medical Center
**NRTI**	Nucleos(t)ide Reverse Transcriptase Inhibitor
**NNRTI**	Non-Nucleoside Reverse Transcriptase Inhibitor
**NVP**	Nevirapine
**PBMC**	Peripheral Blood Mononuclear Cells
**PI**	Protease Inhibitor
**QoL**	Quality of Life
**QALY**	Quality-Adjusted Life Year
**RCT**	Randomized Clinical Trial
**RLS**	Resource-Limited Settings
**TLC**	Thin Layer Chromatography
**UMCU**	University Medical Center Utrecht
**VL**	Viral Load
**WHO**	World Health Organization

Confidential

1 SUMMARY

Rationale

We propose a novel low-cost intensified HIV-1 treatment monitoring strategy that will allow for (1) detection of therapy failure at an early stage, (2) immediate identification of patients with major non-adherence to therapy enabling intensified and tailored adherence counselling, (3) early detection of drug resistance prior to accumulation of resistance mutations, and (4) avoiding treatment switches to second-line regimens when drug resistance is absent. As this strategy is expected to reduce the duration of viremia during therapy failure it is expected to contribute to lower rates of transmission of (drug-resistant) HIV.

Objective

We aim to perform a randomized clinical trial comparing the proposed intensified monitoring strategy with standard of care HIV-1 treatment monitoring in patients who have recently initiated ART. We aim to investigate the benefits and cost-effectiveness of the proposed strategy relative to the standard of care. We will assess the effect on (1) relative risk of development and accumulation of resistance, (2) prevention of unnecessary switches to second-line therapy and (3) resuppression rates after detected viral rebound. We also aim to estimate the effect of the proposed strategy on the transmission of (drug resistant) HIV, and to use trial data to study potential risk factors and determinants of non-adherence and therapy failure.

Study design

An open-label randomised controlled trial.

Study population

HIV-positive men and women, 18 years and older, attending Ndlovu Medical Centre (Elandsdoorn, Limpopo, South Africa) and eligible to initiate or already receiving ART.

Confidential

Inclusion, exclusion and randomisation criteria

Inclusion criteria

- HIV-1 infected male or female patients
- For ART Naïve patients: Eligible for and intending to initiate ART at the clinical site
- For patients on ART: On ART ≥1 year. Last VL <6 months ago and <1000 copies/mL
- ≥18 years of age
- Able to understand and willing to give informed consent

Exclusion criteria

- Any serious unstable medical condition at study baseline
- Any criteria that in the opinion of the investigator indicate that the patient is unable to participate in the full study.

Randomisation criteria

- Completion of six months of continuous ART
- For ART Naïve patients: A VL of <1000 copies/mL at the first VL measurement after initiation at month 6 of ART or a VL of >1000 copies/mL at month 6 of ART followed by a VL of <1000 copies/mL before or at month 9 of ART.
- No signs of active severe opportunistic infection requiring therapy at time point of randomization, excluding infection with *M. tuberculosis*.

Study outcomes

Virological effects of intensified monitoring

- Amount of drug resistance mutations per patient with therapy failure in each arm
- Selection of specific resistance mutations associated with multi-drug resistance in each arm
- Consequential loss of therapeutic options between arms
- Rate of virological failure in the absence of drug resistance between arms
- Rate of resuppression after detectable viremia in each arm
- Unnecessary treatment switches averted in the intervention arm and unnecessary therapy switches made in the control arm

Confidential

- Loss of therapeutic options over time in the presence of a failing regimen in both arms
- The influence of genotypic resistance testing on the choice for a second line regimen in the intervention arm
- Time on a failing regimen per patient with therapy failure in each arm

Clinical effects of intensified monitoring

- Decrease in CD4 count during failure in each arm
- Quality of life in each arm
- Any event of patient morbidity and mortality that can be related to immunological failure in each arm

Feasibility of intensified monitoring

- The practical and logistical implementation challenges
- The time needed to set up the on-site drug level assay
- The realization of expected turnaround times
- The effect of genotyping and drug level assessment results on the choice of second line therapy

Cost-efficiency of intensified monitoring

Comparison of cost increases associated with the intensified monitoring strategy and cost reductions by prevention of unnecessary switches to a more costly regimen and differences in other study outcomes between arms.

Effect on transmission of HIV

Estimation of the effects of the intensified monitoring strategy at the population level by study of the relationships between viremia during therapy, infectivity of patients on treatment and incidence and prevalence of (drug resistant) HIV.

Risk factors for therapy failure

Analysis of the contribution of potential social, economical and psychological parameters to suboptimal adherence, therapy failure, and drug resistance. This will provide the opportunity for development of a scoring model for the risk of therapy failure.

Determinants of therapy failure

In depth studies are planned to gain insight in determinants of therapy failure.

Confidential

Benefit, burden and risks associated with participation

Immediate benefit

Early detection of suboptimal adherence, viremia and drug resistance in patients randomised to the intervention arm, resulting in faster switching of therapeutic regimen in case of drug resistance, avoiding switching in case of no resistance, and in more effective adherence counselling in the case of non-adherence, which are all in the interest of the patient.

Long-term benefit

Contribution to the development of more efficient methods of HIV-1 treatment monitoring for future patients as well as expansion in knowledge of risk factors and determinants of HIV-1 therapy failure.

Risks

Blood sampling: Minimal additional risk. When blood is drawn, patients might experience slight discomfort or possible inflammation at the site where blood is drawn.

Questionnaires: Minimal risk. Participants might find certain questions of a sensitive nature. Referral to a social worker for psychosocial support will be available, if needed.

Confidential

2 INTRODUCTION AND RATIONALE

2.1 Introduction

During recent years access to combination antiretroviral treatment (ART) for HIV-1 infected individuals in low- and middle-income countries (LMIC) has greatly improved. By the end of 2013, 11.7 million people in LMIC were receiving ART, of which the majority lives in sub-Saharan Africa. First-line ART regimens are provided on programmatic basis and usually consist of the Nucleos(t)ide Reverse Transcriptase Inhibitors (NRTIs) tenofovir and emtricitabine or lamivudine combined with a Non-Nucleoside Reverse Transcriptase Inhibitor (NNRTI), efavirenz (EFV). In a meta-analysis of first-line ART in LMIC we demonstrated that, despite high early mortality rates, these strategies are generally successful, with viral suppression rates of 76% after one year and 67% after 2 years of ART.[1]

The initiation of large numbers of patients on lifelong ART commits governments to implement efficient strategies of monitoring of HIV treatment in LMIC. The development of a cost-effective approach to monitoring has been the topic of many recent studies. Several approaches to treatment monitoring have been proposed and implemented in different settings (see table 1). Early trials and modelling studies comparing viral load (VL) and CD4 monitoring with only CD4 monitoring generally concluded that implementation of viral load monitoring in LMIC would bring clinical benefit but at high cost.[2–8] However, these studies generally did not take into account several important consequences of HIV therapy failure, such as ongoing viral transmission due to prolonged undetected viremia and development, accumulation and transmission of drug resistance. Moreover, these studies were performed when prices of VL measurement were substantially higher.[9] In contrast, more recent research has stressed the benefits of implementing routine VL monitoring in LMIC, emphasizing that virological failure is an early indicator that is able to detect therapy failure before its detrimental effects occur.[10–15] These insights are reflected in the 2013 WHO consolidated ART guidelines recommendation stating that, if available, VL testing should be used for monitoring of the effect of HIV treatment and detection of therapy failure.[16] In accordance with World Health Organisation (WHO) guidelines, the South African National Department of Health (NDoH) consolidated treatment guidelines have implemented routine VL monitoring for monitoring of treatment success.[17]

Confidential

Table 1: overview of different monitoring strategies

	Clinical monitoring	Immunological monitoring	Low intensity virological monitoring (2013 WHO guidelines)	High intensity virological monitoring (high-income countries)
Monitoring of symptoms	X	X	X	X
Toxicity monitoring	X	X	X	X
CD4 count		6-12 months	12 months	3-6 months
Viral load			12 months	3-4 months
Genotypic resistance testing				If elevated VL
Drug level monitoring				(on indication)

The frequency of VL monitoring in LMIC such as South Africa is limited to yearly measurements, which is recommended by both WHO and South African NDoH guidelines.[16–18] Upon detection of therapy failure as indicated by a confirmed VL >1000 copies/ml, patients are empirically switched to a second-line regimen containing a boosted protease inhibitor (PI) (ritonavir-boosted lopinavir) and two NRTI’s (commonly zidovudine and lamivudine), and given intensified adherence counselling. However, in previous work we and others demonstrated that the practice of yearly VL monitoring leads to significant accumulation of drug resistance mutations and loss of options for follow-up therapeutic regimens.[15,19] In the same study, it was retrospectively shown that more frequent VL measurement could considerably limit accumulation of resistance by earlier detection of therapy failure followed by switching to a second-line regimen.

Increasing the frequency of VL monitoring may prevent accumulation of resistance but may also result in higher rates of detection of therapy failure in the absence of drug resistance. In previous studies performed in low- or middle income settings of 6 monthly or yearly VL monitoring, 13-35% of patients meeting WHO criteria for therapy failure did not have drug resistance mutations warranting a switch to a second-line regimen.[20–28] In high-income countries this is prevented by combining more frequent VL measurements (usually every three months in the first years of treatment) with genotypic resistance testing in case of viral rebound.[29,30] If drug resistance is detected, a therapy switch to a second-line antiretroviral regimen is made. If drug resistance is not detected, this suggests that viremia is due to non-adherence, and continuation of the first-line regimen combined with intensified adherence counselling is warranted. In some cases, plasma drug levels are measured to confirm whether viremia is a result of non-adherence to therapy.[31] As genotypic resistance testing and drug level monitoring usually require cold chain conditions for sample transport, considerable diagnostic expertise and advanced laboratory equipment, these methodologies are currently

Confidential

not routinely implemented in LMIC.

Delayed detection of therapy failure may have an impact on public health. While it has been shown that levels of ART uptake decrease the risk of HIV transmission on the population level in a South African cohort,[32] such successes may be threatened by frequent occurrence of prolonged therapy failure, which allows for increased transmission rates of (drug resistant) HIV. This is highlighted by a recent WHO survey in Africa that showed that the prevalence of transmitted drug resistance to components of first-line regimens, specifically NNRTIs, has increased after the roll-out of ART.[33] A recent modeling study suggested that high rates of transmitted drug resistance can aggravate HIV epidemics, providing a rationale for close monitoring and prevention of transmission of drug resistance.[34]

As stated by the WHO low-cost and easily implementable alternatives for currently available monitoring tools are urgently needed. In previous research projects we have developed two affordable tools that show the capability to be implemented in clinical treatment programs in LMIC. We and others have developed methods for on-site drug level measurement (DLM) that enables measurement of nevirapine (NVP) and efavirenz (EFV) plasma drug levels at low cost in low income settings requiring minimal laboratory infrastructure.[35–39] Furthermore, we have designed a genotypic drug resistance test optimized for non-B subtypes prevalent in sub-Saharan Africa, which can be performed on dried blood spots (DBS), drastically reducing the cost of and required laboratory infrastructure for drug resistance testing. In a pilot study we have demonstrated that DBS transported at room temperature serve as a good alternative to plasma for drug resistance testing, and can be easily implemented in LMIC.[40]

2.2 Rationale

ART is being rolled out to millions of patients in LMIC. While treatment programmes in LMIC are generally effective, rates of therapy failure, accumulation of drug resistance, unnecessary switching to second-line regimens and (possibly) residual transmission are relatively high. Viral load (VL) monitoring is being implemented in many countries in an effort to improve treatment programme outcomes.[10,41,42] However, the optimal cost-efficient frequency of VL monitoring is currently unknown, and a growing body of evidence suggests that significant individual and population health gains can be achieved by expanding viral load monitoring to genotypic drug resistance testing and drug level monitoring (DLM).[43,44] Conventional tools for resistance testing and DLM are generally not implemented in LMIC due to financial and infrastructural constraints. In pilot projects we have performed field studies using novel low cost solutions for resistance testing and DLM which can be implemented on the individual patient level in LMIC.

Confidential

Meanwhile, price reductions of VL monitoring have added to the affordability of intensified monitoring strategies in LMIC. These recent developments result in a unique opportunity to assess the added value of intensified VL monitoring in combination with drug resistance testing, DLM, and intensified adherence counseling tailored to DLM results in LMIC.

We have developed a novel low-cost strategy for HIV treatment monitoring that enables early detection of therapy failure, drug resistance and non-adherence, while requiring minimal on-site laboratory capacity and being tailored for implementation in settings with monthly medication collection visits. The strategy consists of intensified VL monitoring every three months. In case of a viral rebound >1000 copies/ml, this will be followed up by repeat VL measurement combined with affordable on-site and real time DLM assay targeting NNRTIs. In case NNRTI concentrations are not detected in plasma tailored intensified adherence counselling is performed. In case NNRTI concentrations are detected, this will be combined with off-site DBS-based genotypic drug resistance testing. Switching to a second line regimen will only be performed if drug resistance is detected.

It is expected that intensified VL monitoring will detect therapy failure at an early stage, prior to selection of multiple resistance mutations. In those patients in which viremia is present, DLM is expected to identify patients with (nearly) undetectable plasma drug levels due to major non-adherence, in which the risk of drug resistance is expected to be lower. This will enable clinicians to provide intensified and tailored adherence counselling and prevent unnecessary resistance testing. Effective intensified adherence counselling has the potential to resuppress the VL to below 1000 copies/ml in over half of cases, if performed in an effective manner and in a setting of frequent VL monitoring.[45]

When non-adherence is not detected by DLM, quick follow-up drug resistance testing will detect drug resistance prior to accumulation of resistance mutations and assure safe options for future treatment, preventing the use of more toxic regimens and therefore avoiding additional morbidity. It will also identify those patients in whom drug resistance mutations are absent, thus avoiding unnecessary treatment changes. Finally, as intensified monitoring is expected to reduce the duration of viremia during therapy failure it is expected to lower HIV transmission rates and transmission of drug-resistant HIV. Although the costs of intensified monitoring itself are higher, implementation of the strategy may be cost-effective or even cost saving by preventing unnecessary second-line therapy, reducing patient morbidity and mortality and reducing transmission rates.

Confidential

We aim to perform a randomized controlled trial comparing the proposed novel intensified monitoring strategy with standard of care HIV-1 treatment monitoring, to be performed in adult South African HIV-1 patients who have recently initiated ART. By performing this trial we aim to investigate the benefits and cost-effectiveness of the proposed strategy of intensified monitoring relative to the standard of care. We will assess the effect of the proposed strategy on relative risk of development and accumulation of resistance, prevention of unnecessary switches to second-line therapy and viral resuppression rates. We will also study whether more intensified monitoring has an impact on patient health. Using mathematical modeling, the effect of different monitoring strategy on the transmission of (drug resistant) HIV will be estimated. We aim to collect additional data on sociological, economic, psychological and behavioral risk factors for non-adherence and therapy failure. Investigation of the proposed strategy in a controlled fashion will also allow for future in depth study of immunological, virological and pharmacological mechanisms underlying therapy failure.

Confidential

3 OBJECTIVES

3.1 Primary objective – Virological effects of intensified monitoring

To assess the effect of the intensified monitoring strategy versus standard of care monitoring on (1) development and accumulation of resistance mutations and consequential loss of therapeutic options, (2) prevention of unnecessary treatment switches and (3) resuppression rates after intensified counselling.

3.2 Secondary objectives

3.2.1 Clinical effects of intensified monitoring

To assess the impact of the intensified monitoring strategy on (1) Quality of Life (QoL), (2) patient morbidity and mortality and (3) rates of immunological recovery. Clinical effects will be studied as a secondary objective as it is unknown if the follow-up duration will allow expected differences between arms to become apparent.

3.2.2 Feasibility of intensified monitoring

To assess the feasibility of the intensified monitoring strategy. The results of this study will enable assessment of the feasibility of the intensified monitoring strategy and identify practical and logistical barriers to implementation of the approach.

3.2.3 Effect on transmission of HIV

To model the effects of intensified monitoring on transmission rates of (drug resistant) HIV on a population level, by using a mathematical model incorporating study results such as duration of viremia, presence of drug resistance and sexual risk behaviour as input data.

3.2.4 Cost-effectiveness of intensified monitoring

To assess the cost-effectiveness and incremental cost-effectiveness ratio (ICER) of the intensified monitoring strategy, taking into account the impact on QoL, morbidity and mortality, costs related to individual patient care such as diagnostic interventions, treatment switches and hospital admissions, and cost reductions on a population level as a result of averted new infections.

3.2.5 Risk factors for therapy failure

To gain insight into risk factors for therapy failure and non-adherence by correlating biological and medical parameters such as viral suppression, drug levels, drug resistance,

Confidential

morbidity, mortality and QoL to a validated adherence questionnaire measuring sociological, economic and psychological parameters.

3.2.6 Virological, immunological and pharmacological determinants of therapy failure

To study virological, immunological and pharmacological determinants underlying therapy failure by measuring markers and comparing them between groups of failing and successfully treated patients. (Funding for this objective is not yet available, if obtained, ethics approval will be sought in an amendment to this protocol.)

Confidential

4 STUDY DESIGN AND PATIENT POPULATION

4.1 Study format*

The study format will be an open-label randomized controlled trial (RCT) in which HIV-1 infected patients initiating ART and already on ART will be enrolled. Enrolment will continue until 600 patients have been randomized. Patients initiating ART will be randomized after six months of ART and patients already on ART will be randomised at 6 months after the last viral load measurement. Patients in both arms will receive study visits every three months for a total follow-up duration of 18 months after randomisation to either of two study arms.

- Control arm: Standard of care HIV-1 treatment monitoring in accordance with South African NDOH guidelines.[17] (see “5.1.2”)
- Intervention arm: Intensified treatment monitoring according to the treatment monitoring strategy under investigation. (see “5.1.3”)

4.2 Population

This study will include adult HIV-1 infected patients who are about to initiate or have already initiated first-line ART. Patient enrolment and randomisation will be performed at two different time points. Timing and criteria for enrolment and randomisation are as follows:

1. ART naïve patients: Patients are eligible for enrolment at initiation of treatment with first-line ART. Randomization is performed after availability of the first routine VL measurement, performed at month 6 of treatment.
2. Patients on ART: Patients are eligible for enrolment after ≥ 1 year of virologically suppressive first-line ART and only if a last VL with a result of of <1000 copies/mL was performed within the last 6 months. Randomisation is performed 6 months after the last VL result.

A total number of 600 patients will be randomized into two trial arms. The trial will be conducted on-site at one of the clinical facilities of Ndlovu Care Group (http://www.ndlovucaregroup.co.za/), one of the partners in the project. This facility, Ndlovu Medical Centre, is situated in the town of Elandsdoorn, Limpopo, South Africa, and provides medical service to local South African patients who are unable to pay medical insurance. The

* In this protocol timing of study visits and procedures is indicated in months (defined as a period of 28 days) after the date on which ART was initiated. For the purpose of this protocol, whenever an event is timed at a certain month, a margin of 28 days before and 28 days after the exact date must be maintained. Hence, if a patient has been on ART since September 4th, 2014, his/her month 6 study follow-up visit is due Thursday, February 19th 2015, and will fall within the time frame of Thursday, January 22nd 2015 and Thursday, March 19th 2015.

Confidential

Ndlovu Care Group distributes antiretroviral medication in the framework of the South African Department of Health HAART programme. This clinic is currently providing ART to >3600 patients. Patients on ART return to the clinic monthly for collection of medication, pill count and adherence counselling, which allows intensification of monitoring without substantial change of the infrastructure or frequency of visits.

4.3 Inclusion criteria (study baseline)

- HIV-1 infected male or female patients
- For ART Naïve patients: Eligible for first-line ART and intending to initiate first-line ART at the clinical site
- For patients on ART: On first-line ART ≥1 year. Last VL <6 months ago and <1000 copies/mL
- ≥18 years of age
- Able to understand and willing to give informed consent

4.4 Exclusion criteria (study baseline)

- Any serious unstable medical condition at study baseline
- Any criteria that in the opinion of the investigator indicate that the patient is unable to participate in the full study.

4.5 Randomisation criteria (month 6)

- Completion of six months of continuous first-line ART.
- For ART Naïve patients: A VL of <1000 copies/mL at the first VL measurement after initiation at month 6 of ART or a VL of >1000 copies/mL at month 6 of ART followed by a VL of <1000 copies/mL before or at month 9 of ART.
- Absence of severe active opportunistic infection requiring therapy at time point of randomisation, excluding infection with *M. tuberculosis*.

Confidential

5 DESIGN & METHODS

5.1 Study design

5.1.1 Inclusion, observational follow-up & randomisation (baseline to month 6 of ART)

Eligible patients attending Ndlovu Medical Centre will be recruited as described under “6.2.2 Recruitment procedure” and “6.2.5 Informed consent”. A subgroup of patients is enrolled prior to start of ART in order to collect baseline plasma samples and data on potential risk factors for therapy failure. These patients will receive observational study follow-up visits at month 3 and month 6 after start of ART. In standard of care, at month 6 of ART a VL measurement is performed to determine initial treatment success, indicated by suppression of the VL to <1000 copies HIV-1 RNA/ml. To ensure that only patients with initial treatment success are selected for randomisation, randomisation will be performed after availability of the month 6 VL into either the control arm or intervention arm. (described under “7.2.4 Randomisation procedure”). In addition, a group of patients already on ART and with a last annual VL result of <1000 copies/ml obtained <6 months previously will be enrolled, and will be followed observationally until 6 months after the last viral load, at which point these patients will be randomised in the same fashion. After randomisation, patients in both study arms will return for study follow-up visits on a three-monthly basis, at month 9, 12, 15, 18, 21 and 24 after start of ART or after the last VL measurement. In addition, patients will be called back for additional study visits (max. 2) in case of a detected VL >1000 copies/ml during any of these visits. All visits in both arms, including aforementioned additional call back visits will coincide with standard of care medication collection visits to the clinic. In case of a switch to second-line therapy, patients in both arms will continue three-monthly follow-up visits in an observational manner, and guidelines for monitoring of second-line therapy are followed.[17]

5.1.2 Control arm follow-up – standard of care HIV treatment monitoring

300 patients randomly assigned to this arm will be monitored in full concordance with current South African NDoH guidelines in use at the study site.[17] Viral load measurements will be performed at month 12 and 24 after start of ART (for newly initiated patients) or at month 12 and 24 after the last VL measurement (patients already on ART). If a VL >1000 copies/ml is detected, the patient is called back for counselling for therapy adherence and repeat VL measurement, 2 months after the initial VL measurement. If the repeat VL measurement is >1000 copies/ml after adherence counselling, this is taken to be indicative of therapy failure due to development of drug resistance and a switch to second line therapy is made, together with intensified adherence counselling, without verifying the cause of virological failure by

Confidential

performing DLM or drug resistance testing. If VL drops to <1000 copies/ml after adherence counselling, the first line treatment is maintained. For a full overview of study visits and clinical algorithms in the control arm, see Figure 1: ITREMA study flowchart.

Sample collection in the control arm consists of storage of EDTA-derived plasma and a DBS at study baseline and at the subsequent study visits at month 3, 6, 9, 12, 15, 18, 21 and 24 of ART, as well as during any additional study follow-up visits in case of a VL >1000 copies/ml. Blood draws at baseline, month 3, 6, 12 and 24 after start of ART, and in case of follow-up after a VL >1000 copies/ml coincide with blood draws performed as part of standard of care. The following measurements will be performed on stored samples:

- An additional CD4 measurement at study month 6, in order to assess eligibility for randomisation and rate of CD4-count recovery.
- Retrospective VL testing on stored EDTA-derived plasma in case of clinical therapy failure, in order to retrospectively examine the duration of viremia.
- Retrospective drug resistance testing on stored DBS in case of clinical therapy failure to examine the accumulation of resistance mutations over time.
- Screening for transmitted drug resistance by drug resistance testing on stored DBS obtained as at baseline, prior to start of ART.

5.1.3 Intervention arm follow-up – Intensified HIV treatment monitoring

300 patients randomly assigned to this arm will be monitored using the investigational intensified monitoring strategy. This strategy consists of 3-monthly VL monitoring at month 9, 12, 15, 18, 21 and 24 (after start of ART in initiating patients or after the last VL measurement in patients on ART). If a VL measurement > 1000 copies/mL is detected, the patient will be called back for a follow-up study visit at the next monthly medication collection visit (4 weeks after detection of elevated VL). Upon arrival DLM is performed, the VL measurement is repeated, and a DBS prepared and stored at room temperature. Procedures following this depend on the result of DLM:

- If drug levels are detected by DLM, the result of the VL measurement is awaited. If the repeat VL is >1000 copies/ml, the DBS is shipped directly by courier to the WHO reference laboratory for drug resistance testing. The reference laboratory will provide feedback by means of a digital resistance report to the coordinating research physician. The patient will be called back for a second follow-up study visit at the next monthly medication visit (8 weeks after detection of elevated VL), either for prescription of second-line therapy or continuation of first-line therapy, guided by the

Confidential

result of resistance testing.

- If DLM at the first follow-up visit indicates that drug levels are not detected, intensified counselling is performed at the same visit and first-line therapy will be maintained, regardless of the result of the repeat VL measurement. The patient will not be called back and the next VL will be performed at the next scheduled three-monthly time point. However, if the VL result at this visit is again >1000 copies/ml, drug resistance testing will be performed regardless of the outcome of DLM.

For a full overview of study visits and clinical algorithms in the control arm, see Figure 1: ITREMA study flowchart.

Sample collection in the control arm consists of storage of EDTA-derived plasma and a DBS at study baseline and at the subsequent study visits at month 3, 6, 9, 12, 15, 18, 21 and 24 of ART, as well as during any additional study follow-up visits in case of a VL >1000 copies/ml. Blood draws at baseline, month 3, 6, 12 and 24 after start of ART, and in case of follow-up after a VL >1000 copies/ml coincide with blood draws performed as part of standard of care. The following measurements will be performed on stored samples:

- An additional CD4 measurement at study month 6, in order to assess eligibility for randomisation and rate of CD4-count recovery
- Retrospective drug resistance testing on stored DBS in case of clinical therapy failure to examine the accumulation of resistance mutations over time.
- Screening for transmitted drug resistance by drug resistance testing on stored DBS obtained as at baseline, prior to start of ART.

Confidential

Figure 1a: ITREMA study flowchart – enrolment to randomisation

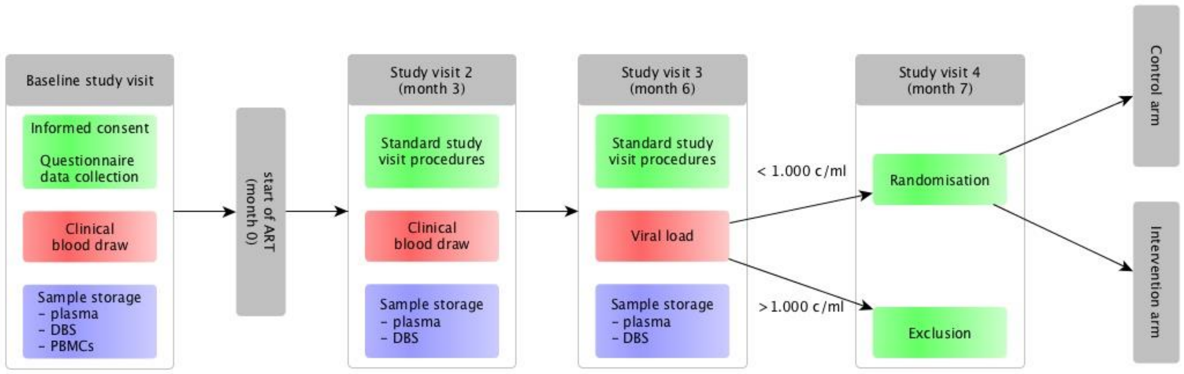

Figure 1b: ITREMA study flowchart – post randomisation

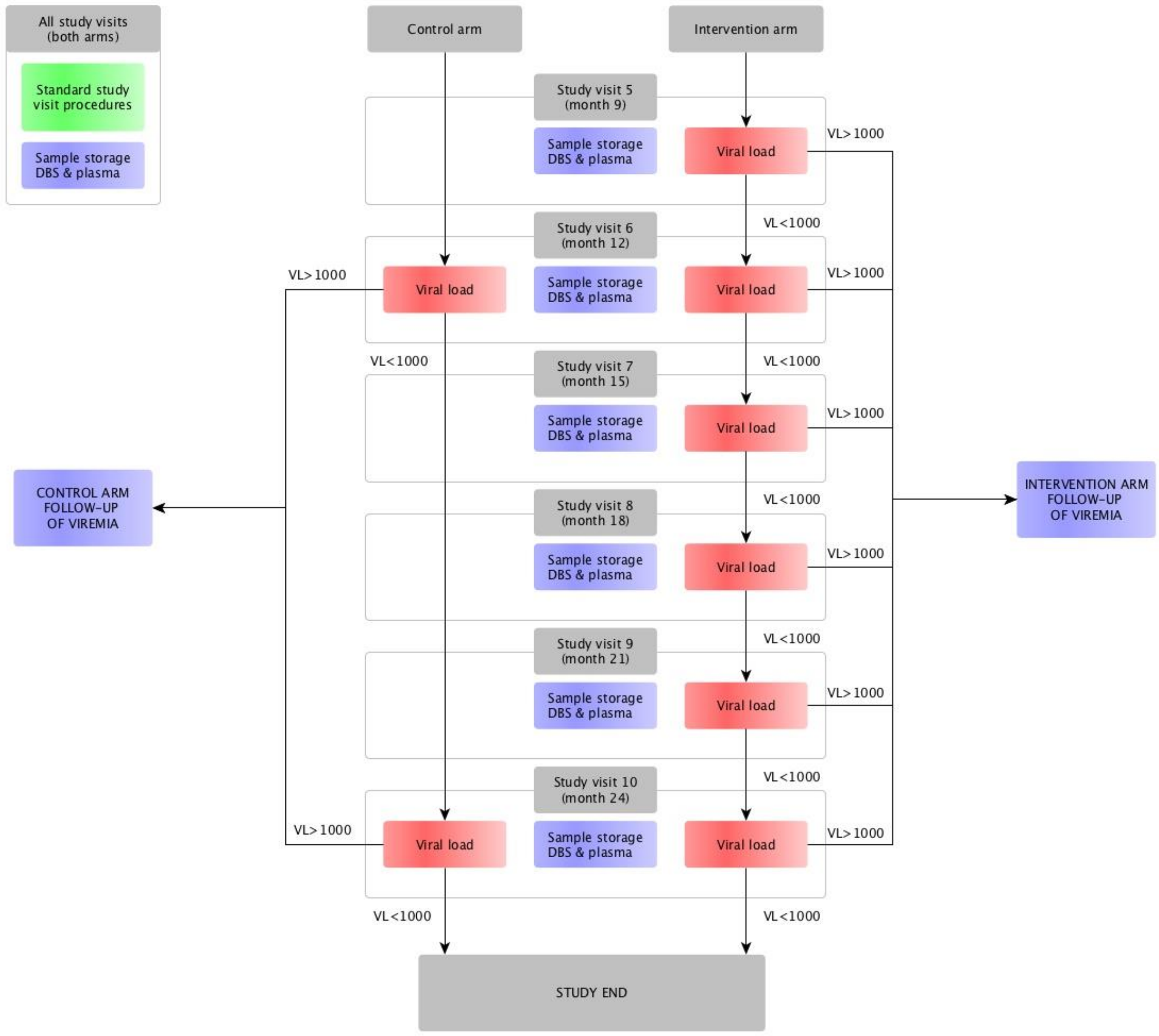

Figure 1c: ITREMA study flowchart – follow-up of viremia in both arms

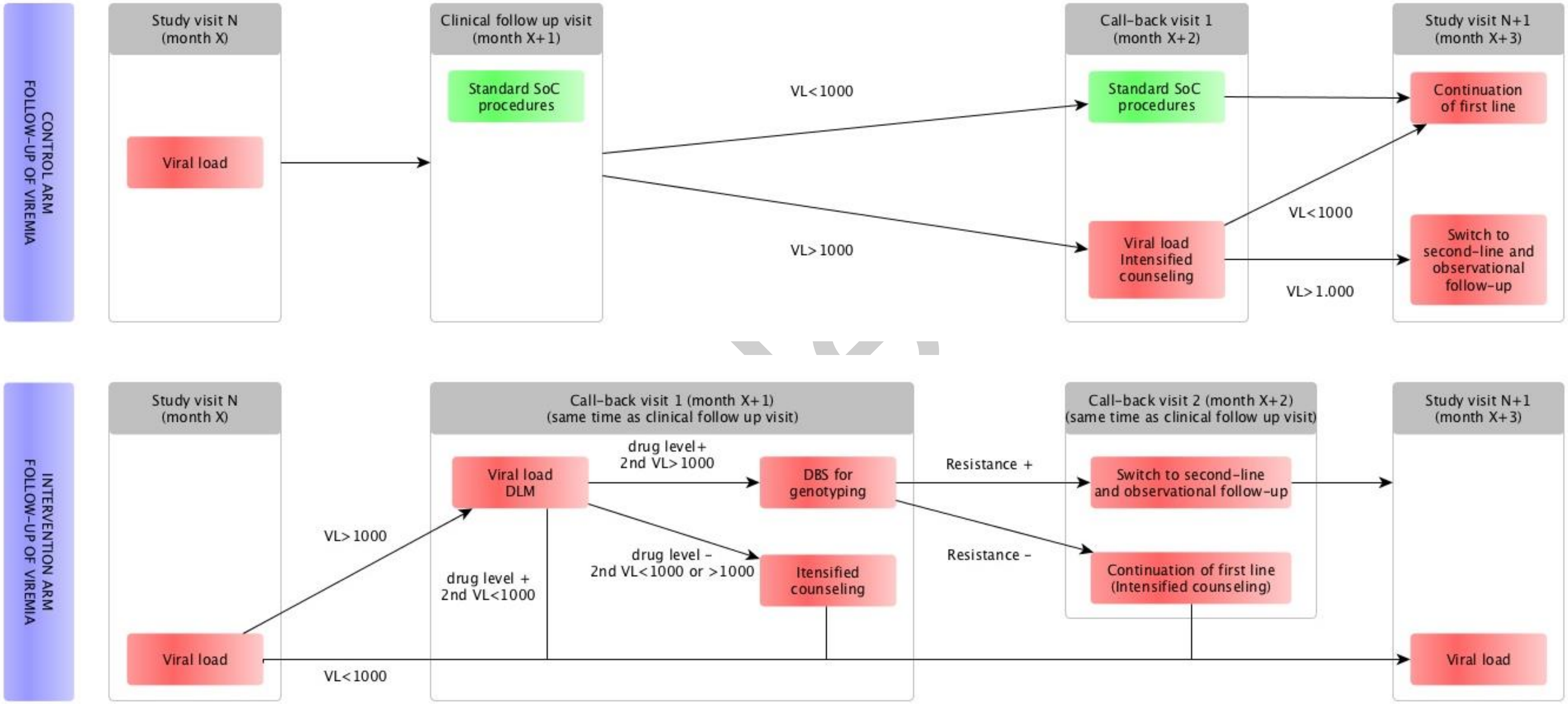

Legend to flowcharts: **plasma**: EDTA-derived plasma, **DBS**: Dried Blood Spot, **PBMC**: Peripheral Blood Mononuclear Cell, **VL>1000**: Viral Load of 1000 copies/ml or more, **VL<1000**: Viral Load of less than 1000 copies/ml, **DLM**: Drug Level Measurement, **drug level +**: detectable concentrations of the NNRTI in plasma, **drug level -**: no dected NNRTI in plasma, **Resistance +**: Drug resistance mutations detected, **Resistance -**: No drug resistance mutations detected.

5.2 Methods and procedures

5.2.1 Laboratory setting

Ndlovu Medical Centre (NMC) has an on-site basic laboratory which is fully equipped with centrifuge, flow hood, -80 and -120 freezers for storage of samples equipped with digital thermo control system, automatic electrical failure alarm system and reconnection to an alternative power supply in case of power cuts. The laboratory personnel are capable of performing CD4 cell count assays and preparation of dried blood spots.

5.2.2 Viral load determination

Quantitative measurement of plasma HIV-1 RNA (viral load) will be determined using a CE/IVD certified kit-based HIV-1 viral load assay, according to the instructions of the manufacturer. Batch wise retrospective analyses will be performed on stored plasma samples of patients with therapy failure in the control arm in order to determine duration of viremia. Results will be reported in copies/ml.

5.2.3 Clinical assessment

A clinical assessment will be performed in both arms, in accordance with the recommendations for "Baseline and routine clinical and laboratory assessment for late adolescents" specified in the South African NDoH HIV treatment guidelines.[17] Separate algorithms are followed for baseline and follow-up visits.

- **Baseline clinical and laboratory assessment** consists of WHO clinical staging, screening for symptoms suggestive of infection with *M. Tuberculosis*, other opportunistic infections and symptoms of concomitant chronic diseases and sexually transmitted infections. Screening of laboratory markers is performed to assess contra-indications to components of first-line ART and, if indicated, for detection of AIDS-defining conditions.
- **Follow-up clinical and laboratory assessment** consists of WHO clinical staging, screening for symptoms suggestive of *M. Tuberculosis* infection and other opportunistic infections.

In addition, weight, co-medication, time of medication intake and other events occurring during clinical follow-up will be recorded. Baseline and follow-up assessments will be recorded in a data collection form. Appropriate clinical action will be taken if abnormalities are detected.

Confidential

Detected abnormalities can form a motivation to perform VL testing outside of the study protocol in conformation with South African NDoH guidelines.

5.2.4 Follow-up of elevated viral load measurement

5.2.4.1 Intervention arm

In case of a VL >1000 copies/ml of a patient in the intervention arm, feedback of this result will be given by the clinical site laboratory after 2-5 working days). The patient will be notified to report back to the research clinician or nurse at his/her next monthly routine clinical visit for medication collection (day 28 after study visit). At this visit repeat viral load testing, DLM and collection of plasma and DBS is performed. If DLM indicates no detectable drug level (see “5.2.5 Drug Level Monitoring”), the patient is informed of this result, and intensified adherence counselling is performed. If DLM does not indicate non-adherence, standard of care adherence counselling will be performed. The result of the second VL will be available after 2-5 working days (day 31-33 after study visit). In case of a second VL >1000 copies/mL and a detectable drug level at DLM, the DBS will be sent to the WHO reference laboratory for drug resistance testing and patients will be notified again to report back to the research clinician or nurse at his/her next monthly routine clinical visit (day 56 after study visit) for genotype guided switch to second-line therapy or for genotype guided continuation of treatment combined with counselling in case no relevant resistance is detected. In case DLM detected no drug level, the DBS is not sent and genotyping not performed. The patient will not be called back and not be switched to second-line therapy prior to the next study visit. In case an elevated VL is again detected at the subsequent study visit (day 84 after previous study visit), a DBS is sent directly for drug resistance testing and genotype guided therapy switch or continuation is performed. In case of continuation, the three monthly monitoring frequency is maintained. In case of switch, a VL will be performed six months after the switch to evaluate initial therapy success. Three monthly study visits will be continued in an observational manner, at which storage of DBS and plasma is performed.

5.2.4.2 Control arm

In case of a VL >1000 copies/ml of a patient in the control arm, VL will be repeated, and intensified adherence counselling will be given during the next routine drug pick-up and counselling visit (2 months after detection). If a second VL is also elevated >1000 copies/ml, switching to second-line therapy is performed and intensified counselling is maintained. If the second VL is <1000 copies/ml, the current regimen is maintained. In case of switch, a VL will be performed six months after the switch to evaluate initial therapy success. Three monthly

Confidential

study visits will be continued in an observational manner, at which storage of DBS and plasma is performed.

5.2.5 Drug Level Monitoring (DLM)

DLM will be performed in case of a VL >1000 copies/ml at any of the intervention arm study follow-up visits. DLM will take place at a call back visit one month after the visit in which elevated VL was detected, using EDTA-derived plasma obtained at the call back visit. DLM will be performed on-site using a qualitative assay that can be implemented in basic clinical laboratories, and will detect plasma levels of the NNRTI's efavirenz and nevirapine. Results will be reported as either "detectable drug level" or "no detectable drug level", the latter indicating at least several days of non-adherence over the last week. This result will be reported back to the treating research clinician at the call back visit, who will inform the patient of the result during this visit.

5.2.6 Dried Blood Spot-based Drug Resistance Testing

In case of a confirmed detectable viral load (>1000 copies/ml) in the presence of detectable drug level as indicated by DLM, a dried blood spot (DBS) sample acquired during the second viral load measurement will be sent to a WHO reference laboratory for drug resistance testing, where genotypic HIV-1 drug resistance testing will be performed using an RT-only assay optimized for non-B HIV subtypes. This assay has been validated for use on DBS and implemented in a pilot study at Ndlovu Medical Centre.[27] The assay consists of a single-round one-tube RT-PCR to amplify the viral RT-gene between codons 40-238, followed by population sequencing of the amplified fragment in both directions using 2 primers only. With this approach all RT resistance information relevant to first-line regimens is generated at minimal cost for laboratory reagents, equipment and personnel.[46] If resistance testing is indicated in patients using second line therapy, a protease-only genotypic assay will be performed on the DBS in addition to the RT-only assay.[27] Drug resistance testing will also be performed retrospectively on stored DBS in all patients with therapy failure in the control group, using the same approach as described above. The WHO reference laboratory will provide the treating physician with the result and interpretation of the drug resistance test, containing viral subtype, a list of all mutations, specific indication of relevance of these mutations to drug-resistance, a drug susceptibility ranking using resistance interpretation algorithms, and an overall interpretation with treatment advice taking local availability of antiretroviral drugs into account.

5.2.7 Questionnaires

Confidential

Questionnaires will be used to measure a variety of parameters relevant to the trial study objectives (see table 2). For data on adherence (e.g. number and reasons of missed doses, reasons and motives for non-adherence), drug toxicity and physical and mental wellbeing, a questionnaire developed by the AIDS Clinical Trial Group (ACTG) will be used. This validated self-report questionnaire has been included in several clinical trials and consists of a baseline and follow-up questionnaire. A modification of the ACTG baseline questionnaire will be used at enrolment, and a shortened version of the ACTG follow-up questionnaires will be used at month 3, 6, 9, 12, 15, 18, 21 and 24 (study end) in both study arms. Data on adherence from the standard pill count form that is applied at each drug pick-up visit (monthly or every 2 months) will also be collected.

Data relevant to the study objectives regarding social and psychological risk factors are partly covered by the ACTG questionnaires. A newly designed questionnaire will be used that will incorporate the sections from the ACTG questionnaires (mental and physical health), NIDS (socioeconomic status and family structure), CHAMP (substance abuse and sexual risk behavior). This questionnaire will contain item scales modified from the ACTG original, matched to local socio-economic and cultural context, as well as record several additional parameters such as stigmatization (Kalichman & Simbayi) and coping abilities (CISS). Data on Quality of life (QoL) of study participants will be used for evaluation of the cost-effectiveness of the trial intervention and will be recorded using the WHO QOL HIV (BREF) questionnaire and EQ5D questionnaire.

In a substudy participants who become lost to follow-up during the trial and who are successfully traced >28 days after a missed clinical visit (as per standard-of-care clinical procedures) are approached for informed consent to perform an additional questionnaire interview during a home visit.

Questions will be bundled in a comprehensive baseline and study end questionnaire (baseline and month 24), and a brief follow-up questionnaire which will be recorded per 3-monthly study visit (month 3, 6, 9, 12, 15, 18 and 21). In lost to follow-up patients who enter the substudy questionnaires will be recorded during a home visit. For a full overview of parameters and questionnaires, see table 2.

Confidential

Table 2: overview of questionnaire data

**Parameters**	**Time of collection**	**Source**
Socio-economic status<br>- Demographics (1)<br>- Employment (2)<br>- Household composition (3)<br>- Food insecurity (4)	Baseline	- NIDS wave 3 Adult questionnaire<br>- NIDS wave 3<br>- NIDS wave 3<br>- SAHANES
Health<br>- Mental health (5)<br>- Physical health (6)	Baseline & study end	- PHQ-9
Transmission factors<br>- Sexual risk behaviour (7)<br>- Transactional sex (8)	Baseline	- CHAMP<br>- CHAMP
Adherence factors<br>- Attitudes toward therapy (10)<br>- Stigmatization (11)<br>- Coping abilities (12)<br>- Substance abuse (13)	 Baseline<br>Baseline<br>Baseline<br>Baseline	 - ACTG Baseline & FU<br>- Kalichman&Simbayi<br>- CISS-21<br>- CHAMP
Pill counts and<br>self-reported adherence (SoC)	Baseline & every 3 months<br>home visit in LTFU patients	South African NDoH standard of care pill count form
Quality of life<br>- HIV-related QoL<br>- QoL for QALY valuation	Baseline & every 3 months	- WHO QOL HIV (BREF)<br>- EQ5D
Substudy loss to follow-up (LTFU)<br>- Outcomes after LTFU<br>- Patient-reported reasons for LTFU	Phone call & home visit in LTFU patients	- Wilkinson, Skordis et al (2015)<br>- Tweya, Feldacker et al (2013)<br>- Geng, Odeny et al (2015)

Confidential

5.2.8 Determinants of therapy failure

In all patients peripheral blood mononuclear cells (PBMCs) will be isolated at the baseline study visit, prior to start of therapy. The availability of stored PBMCs and plasma at baseline will enable future study of virological, immunological and pharmacological determinants of therapy failure in those patients in which therapy failure occurs. During study follow-up PBMCs will be isolated every three months in a subgroup of patients. Permission for this sub study will be requested in an amendment to the ethics committee.

5.3 Study visits and procedures per visit

5.3.1 Visits

In the paragraphs below the activities during the different study visits are described. For every activity, it is listed in **bold** whether the research counsellor, **research nurse** or **research clinician** performs the activity. At least one of these will be fluent in local languages, enabling consultation in native languages in all cases. A full overview of study visits is shown in table 3.

5.3.1.1 Baseline visit

Participants will be enrolled at a regular clinic visit. During this visit, patient informed consent will be obtained by the **research counsellor**. After informed consent a questionnaire interview will be performed by the **research counsellor** who will explain and translate all questions if necessary and who will record all baseline questionnaire data ("5.2.7 Questionnaires") on a digital system. Blood will be drawn for storage of EDTA-derived plasma, preparation of DBS and isolation of PBMCs (**research nurse**) and a physical examination will be performed (**research nurse or clinician**). Clinical data will be recorded in a data collection form ("5.2.3 Clinical assessment").

5.3.1.2 Randomisation

At the month 6 study visit, a VL measurement will be performed as part of standard of care. Between the month 6 and month 9 study visit participants will be informed of the VL result and, if applicable, informed of the randomisation procedure and of their randomisation result (see "6.2.3 Randomisation procedure") by the **research nurse** or **research clinician**. If randomisation criteria are not met, either due to viremia >1000 copies/ml or presence of severe clinical conditions, the patient will be informed of these results and excluded from the study, a health assessment will be performed by the **research clinician**, and the patient will be transferred back to clinical care and offered referral to a health care provider of their choice.

5.3.1.3 Follow-up visits

Study follow-up visits in both arms will occur at month 3, 6, 9, 12, 15, 18, 21 and 24 after initiation of ART or after the last VL measurement. These visits will be planned to coincide with monthly drug pick-up visits to ensure that no extra visits to the clinic are needed. At each visit a follow-up questionnaire interview is held ("5.2.7 Questionnaires") by the **research counsellor**, a medical and physical examination is performed and blood is drawn for storage of EDTA-derived plasma and preparation of DBS (**research nurse**). In case of clinical symptoms or compliations the **research clinician** will be consulted. Clinical data will be recorded ("5.2.3 Clinical assessment").

5.3.1.4 Call-back in case of elevated viral load

Intervention arm:

A follow-up VL measurement will be performed, patients will be notified of the result of DLM (**research nurse**) and adherence counselling will be intensified (**research counsellor**) if DLM

Confidential

and/or pill counts indicated suboptimal adherence. The **research clinician** will be consulted if symptoms are noted by the **research nurse**.

Control arm:

A follow-up VL measurement will be performed (**research nurse**) and adherence counselling will be intensified (**research counsellor**) if pill counts indicate suboptimal adherence. The **research clinician** will be consulted if symptoms are noted by the **research nurse**.

5.3.1.5 Call-back in case of second elevated viral load

Intervention arm:

If DLM indicated a detectable drug level at the previous visit and relevant drug resistance mutations are present, a second-line regimen will be prescribed during this visit (**research clinician**). In case DLM indicated no detectable drug level, drug resistance testing is not performed, the patient will be notified of the results (**research nurse**) and will again receive additional adherence counselling at the next study visit (**research counsellor**).

Control arm:

Patients will be switched to second-line therapy (**research clinician**) and adherence counselling is intensified (**research counsellor**).

5.3.1.6 Immediate tracing after a missed visit

In case of a missed scheduled study visit by a study participant, local standard-of-care clinical retention procedures will initially be followed in both trial arms. Patients receive an SMS test message two days after a missed appointment, a phone call if they do not return after the text message (~4 days after missed visit) and a home visit if they do not respond to the phone call and live within the clinic catchment area (~1-2 weeks after missed visit). If retrieved, they are then counselled to attend the clinic as soon as possible for a study visit.

5.3.1.7 Tracing 28 days after a missed visit

As per clinical standard-of-care, patients who do not return to the clinic within 28 days after a missed scheduled appointment will receive a phone call and questionnaire on current location and contact details and treatment status is administered. If telephonic contact cannot be made a home visit is performed when the patient has consented to this. During home visits the standard of care questionnaire on current location and contact details and treatment status is administered.

5.3.1.8 Immediate tracing after a missed visit

Confidential

In addition to standard-of-care and if informed consent is given, tracing procedures in study participants are expanded by performing home visits in all participants regardless of successful telephonic tracing, during which an in-depth questionnaire interview on reasons for LTFU and a finger prick for sampling of a DBS is performed.

As per standard-of-care, 90 days after a missed scheduled appointment retention status will be re-evaluated. Patients not returned to the clinic will be contacted telephonically and per home visit in case of no telephonic contact to confirm their current treatment status. At this time point the definite patient study outcome will be recorded.

If not retrieved, a patient will be reported as lost to follow up and excluded if two subsequent study visits are missed and the retention procedure for both visits is unsuccessful. If ART is stopped or interrupted, patients will also be excluded from the study. Study exclusion will in no way compromise access to medical care.

5.3.1.9 Clinical (symptoms) monitoring and referral in case of symptoms or complaints

Study patients will receive clinical (symptoms) monitoring in line with South African NDoH guidelines, as described above (“5.2.3 Clinical assessment”). All patients with a last recorded CD4 count below 200 cells/mm3 will be monitored by a physician initially and by **the research nurse or clinician** at each subsequent study visit, while all patients with CD4 counts above 200 cells/mm3 will be monitored by **the research nurse** at each study visit. If symptoms are detected by **the research nurse** the patient will be referred to **the research clinician**, who will document symptoms and will perform a physical exam.

If any medical conditions requiring additional treatment are detected at entry into the study or during follow-up contact moments, participants in both control and intervention arms will be attended to and offered referral to a health care provider of their choice.

5.4 Laboratory procedures and storage of blood

A full overview of study blood draws and subsequent sample storage per visit is shown in table 4.

Laboratory tests and procedures (both standard of care and additional measurements in study subjects) will be performed at the on-site Toga laboratories facility (accreditation by the South African National Accreditation System (SANAS)). Genotypic drug resistance testing will be performed by the WHO reference laboratory. All laboratory procedures are described in a Laboratory Manual.

Confidential

Informed consent will be obtained for storage of samples. In case additional research questions are formulated on the stored plasma samples, the Ethics Committee will be asked for additional approval.

5.5 Withdrawal of individual participants

Participants will be informed that they can leave the study at any time for any reason if they wish to do so without any consequences. When a participant decides to leave the study, he/she will be asked for written permission if he/she, or a contact person, can still be contacted in order to gather morbidity/mortality follow up data.

5.6 Replacement of individual subjects after withdrawal

Participants who withdraw from the study during the recruitment phase will be replaced until the intended numbers of inclusion at randomization (n=600) have been achieved.

Confidential

Table 3: Overview of ITREMA study visits – Clinical procedures and Questionnaire data collection (standard of care arm and intervention arm)

	Screening	Baseline	Month 3	Month 6	Month 7		Month 9	Month 12	Month 15	Month 18	Month 21	Month 24	Control Arm Follow-up		Intervention arm follow-up			
**Clinical procedures**					If VL <1000	If VL >1000							If single VL>1000	If twice VL>1000	If single VL>1000	If twice VL>1000; DL+	If twice VL>1000; DL-	If resistance
Informed Consent & Eligibility Check	**X**																	
Randomization					**X**													
Exclusion						**X**												
Medical History		**X**																
Physical Exam/Vital Signs		**X**																
PE if symptomatic			**X**	**X**	**X**	**X**	**X**	**X**	**X**	**X**	**X**	**X**	**X**	**X**	**X**	**X**	**X**	**X**
Clinical Outcomes		**X**																
Concomitant Medications		**X**																
Switch to second-line ART														**X**				**X**
TDM-based counselling															**X**		**X**	
Health care worker (P=physician, N=nurse, C=counselor)	**P/N/C**	**P+N**	**N**	**N**	**N**	**P**	**N**	**N**	**N**	**N**	**N**	**N**	**N**	**P+N**	**N**	**P+N**	**P+N**	**P**
**Questionnaire data**																		
transmission/adherence factors, SES		**X**										**X**						
Health, QoL, self reported adherence		**X**	**X**	**X**			**X**	**X**	**X**	**X**	**X**	**X**						

Legend: IA: Intervention arm, CA: Control arm. CD4: CD4+ T-lymphocyte count. DBS: Dried blood spot. DL-: subtherapeutic drug level. DL+: therapeutic drug level. EDTA: Ethylenediaminetetraacetic acid-containing vacutainer tube. FBC: Full blood count. PE: Physical exam. QoL: Quality of life. SES: Social-economic status. SST: Sodium-seperating vacutainer tube. TDM: Therapeutic drug monitoring. VL: Viral load determination.

Confidential

Table 4: Overview of ITREMA study visits – Laboratory procedures and Blood draws (standard of care arm and intervention arm)

	Baseline	Month 3	Month 6	Month 7		Month 9	Month 12	Month 15	Month 18	Month 21	Month 24	Control arm follow-up of viremia		Intervention arm follow-up of viremia			
**Laboratory procedures**				If VL <1000	If VL >1000							If single VL>1000	If twice VL>1000	If single VL>1000	If twice VL>1000; DL+	If twice VL>1000; DL-	If resistance
Viral Load (CA = Control Arm, IA = Intervention Arm)			**CA+IA**			**IA**	**CA+IA**	**IA**	**IA**	**IA**	**CA+IA**	**CA**		**IA**			
CD4			**X**				**X**				**X**						
DBS and plasma storage (1 EDTA 9ml)	**X**	**X**	**X**			**X**	**X**	**X**	**X**	**X**	**X**	**X**	**X**	**X**	**X**	**X**	**X**
PBMC isolation (2 EDTA 9ml)	**X**																
therapeutic drug monitoring (TDM)														**X**			
DBS-based resistance testing															**X**		
Other standard of care blood tests	**X**	**X**	**X**				**X**				**X**	**X**	**X**	**X**	**X**		
**Blood draw Volume/vacutainer type**																	
EDTA 4ml	**0**	**0**	**1**	**0**	**0**	**0**	**1**	**0**	**0**	**0**	**1**	**0**	**0**	**0**	**0**	**0**	**0**
EDTA 9ml	**3**	**1**	**1**	**0**	**0**	**1**	**1**	**1**	**1**	**1**	**1**	**1**	**1**	**1**	**1**	**1**	**1**
DBS	**1**	**1**	**1**	**0**	**0**	**1**	**1**	**1**	**1**	**1**	**1**	**1**	**1**	**1**	**1**	**1**	**1**
SST 4ml	**1**	**1**	**1**	**0**	**0**	**0**	**1**	**0**	**0**	**0**	**1**	**1**	**1**	**1**	**1**	**0**	**0**
Total blood (mls)	**31**	**13**	**17**	**0**	**0**	**9**	**17**	**9**	**9**	**9**	**17**	**13**	**13**	**13**	**13**	**9**	**9**

Legend: IA: Intervention arm, CA: Control arm. CD4: CD4+ T-lymphocyte count. DBS: Dried blood spot. DL-: subtherapeutic drug level. DL+: therapeutic drug level. EDTA: Ethylenediaminetetraacetic acid-containing vacutainer tube. FBC: Full blood count. PE: Physical exam. QoL: Quality of life. SES: Social-economic status. SST: Sodium-seperating vacutainer tube. TDM: Therapeutic drug monitoring. VL: Viral load determination.

6 OUTCOMES AND ANALYSIS OF RESULTS

6.1 Virological and clinical effects of intensified monitoring

Virological outcomes will be differences between arms in (1) the number of drug resistance mutations present, (2) the selection of specific resistance mutations associated with multi-drug resistance (presence of the K65R mutation, or the presence of >1 NNRTI mutation) and (3) consequential loss of therapeutic options. Furthermore, (4) the rate of virological failure in the absence of drug resistance in both arms, unnecessary treatment switches averted in the intervention arm and unnecessary therapy switches made in the control arm will be assessed. An averted case of unnecessary switching will be defined as two sequential elevated VL results in the intervention arm without presence of resistance mutations. Assessment of unnecessary switching in the control arm can be ascertained by retrospective genotypic resistance testing.

Additional virological outcomes will be (5) the time on a failing regimen, defined as the time during which viremia > 1000 copies/mL is maintained after the first instance of a viral load >1000 copies/ml (6) The loss of second line therapeutic options over time in the presence of a failing regimen. Comparison of the extent of drug resistance between both groups will allow for assessment of the clinical consequences of resistance development in both arms. (7) The influence of genotypic resistance testing on the choice for a second line regimen. Genotypic resistance testing results enable the physician to make an informed decision with regard to a follow-up regimen to the first-line regimen. By comparing the chosen regimens in the intervention and control groups, the effect of the availability of genotypic resistance data on clinical decision-making will be compared. These outcomes can be compared between both arms as VL and CD4 measurements will be performed retrospectively in the control group, resulting in an equal frequency of measurements between both arms.

Clinical outcomes will be decrease in CD4 count during failure, QoL as measured using a validated QoL questionnaire, and recorded events of patient morbidity and mortality that can be related to immunological failure.

6.2 Feasibility of intensified monitoring

With the results of this study, it will be possible to assess the feasibility of intensified monitoring in LMIC and identify practical and logistical barriers to implementation of the approach in low- and middle-income countries. Several outcomes will be studied: (1) The practical and logistical implementation challenges as reported by involved research health care

Confidential

workers. (2) The time needed to set-up and perform a basic local validation of the on-site drug level assay (3) The realization of expected turnaround times, specifically the turnaround time of on-site testing (drug-level measurement) and centralized testing (more frequent viral load measurements and drug resistance testing on DBS). (4) The effect of genotyping and drug level assessment results on the choice of second line therapy, in which it will be determined if the increased availability of information regarding treatment is interpretable and useable in LMIC clinical practice by interviews of the involved medical personnel (physicians and clinical nurses). In addition the time to report of results and actual clinical intervention will be evaluated. The overall results of this evaluation will be to inform policy makers both in South Africa and abroad. Scientific peers and laboratory staff will be instructed on pitfalls and successful approaches.

6.3 Cost-effectiveness of intensified monitoring

The cost increases associated with the intensified monitoring strategy will be compared with the (estimated) cost reductions by prevention of unnecessary switches to a more costly regimen and differences in other study outcomes between arms. To evaluate the cost-effectiveness of the intensified monitoring strategy we will assess the effects of the intensified monitoring strategy on reduction of resistance accumulation and unnecessary treatment switches in a model-based approach with several levels of detail. The first assessment will concern the net cost of implementing tailored patient management based on DBS for resistance genotyping. A Markov model will be developed to estimate the costs over a fixed time period following initiation of ART. Secondly, we will assess the cost-effectiveness of implementing decentralized drug level testing. Here, we will extend the Markov model with known evidence on health outcomes in South Africa, i.e. quality of life using a standardized item scale and mortality. With the extended model the costs and effects, in terms of life-years and quality-adjusted life-years (QALYs) gained, as well as the incremental costs and ICER of (different components of) the intensified monitoring strategy will be estimated. Finally, we will analyze cost-effectiveness from the societal perspective and take indirect costs and benefits into account, e.g. costs and benefits that are generated by the impact of treatment on patients' productivity and participation in society. To fully take indirect effects into account dynamic transmission modeling is necessary.[47] Sensitivity analyses will be performed in order to ascertain how the (cost)-effectiveness of various monitoring schemes depends on behavioral parameters, on treatment uptake and adherence, and other key model parameters.

Confidential

6.4 Effect on transmission of HIV

Using a transmission dynamic model we will assess the effects of monitoring strategies and reduction in resistance development at the population level by studying the relationships between the monitoring strategies, infectivity of patients on treatment and HIV incidence and prevalence. We will design a model that is structured by age and sex, and that explicitly takes CD4+ counts and viral load into account. The model will be calibrated to HIV prevalence in the study region using data available through reports from the South African NDoH.[48] We will estimate parameters pertaining to diagnosis rates, uptake of treatment, adherence to treatment and monitoring from the data collected during this study. We will then analyse various monitoring and treatment scenarios to analyse the impact of different monitoring schemes on HIV incidence and prevalence in the future. Model output on incidence stratified by age and sex can then be used as input for the Markov model for cost-effectiveness assessment. We will also study the possible spread of resistant strains in a population with a high prevalence of treatment sensitive strains. We will assess how spread of resistant strains may depend on treatment uptake and adherence parameters, and how transmission of resistant strains impacts on the (cost)-effectiveness of specific monitoring schemes. Particular focus will be on how to design a monitoring scheme that minimizes the risk of spread of resistant strains in the population while still being feasible and cost-effective. We will study which parameters are key for such a scheme and how it depends on disease specific characteristics such as the infectivity during early HIV infection and heterogeneity in the population with respect to sexual risk behavior and testing behavior. Short and long-term impact on HIV incidence will then be associated with impact on disease burden in the population and with cost-effectiveness of those monitoring strategies.

6.5 Risk factors for therapy failure

By multivariate (regression) analysis of biological parameters such as viral suppression, drug levels and drug resistance social, economical and psychological risk factors, indicators of suboptimal adherence and therapy failure will be ascertained. Data on stigmatization and coping strategies will be used to assess non-adherence risks. These data in their turn will be analysed in order to determine whether adherence is a mediating or moderating factor in defining the patient health outcomes (mental and physical health and QoL). In addition, multivariate analysis of the relationship between risk factors, adherence, patient health and QoL will provide the opportunity for local validation of these item scales and for development of a scoring model for the risk of therapy failure.

Confidential

6.6 Determinants of therapy failure

In depth studies of virological, immunological and pharmacological markers on stored PBMCs and plasma are planned to gain insight in virological, immunological and pharmacological determinants underlying therapy failure. Detailed outcomes that will be measured as part of these sub studies will be described in an amendment to the current protocol.

6.7 Statistical methods

6.7.1 Analysis of clinical study outcomes

Study data that has been captured into a secured database (see "7.1.1 Data collection") will be exported and loaded into a statistical software programme for statistical analysis without any presence of any personal identifying information.

6.7.2 Analysis of clinical study outcomes

In univariate analysis on dichotomous variables the χ^2-test will be used. Continuous variables will be analysed using the Student t-test or Mann-Whitney U test. For dichotomous primary outcome measures the relative risk, absolute risk and risk difference will be reported. Continuous variables will be summarized by medians with interquartile range and means with standard deviations. For univariate analysis on continuous outcomes mean differences will be reported. For survival analysis the Cox proportional hazards model will be used, and hazard ratio's will be reported. Assessment of risk factors for therapy failure as well as its potential interaction with patient health and QoL will be performed using multivariate analysis. All results will be reported along with 95% confidence intervals.

6.7.3 Sample size calculation

Sample size calculation is based on power analyses using selection of resistance as the primary study outcome. The first outcome used for power calculation is the selection of non-polymorphic K65R drug resistance related mutation in HIV reverse transcriptase that is selected by currently used first line therapy at any time during study follow-up. Previously observed prevalence rates of the K65R mutation in HIV-1 subtype C infected patients experiencing therapy failure of a first-line regimen are 70% in LMIC and 17% in developed settings.[49,50] Using a 1-α of 0.9 and a β of 0.05, the inclusion of 14 cases of failure per arm is needed. Cumulative virological failure (VL >1000 copies/ml) is anticipated to occur in 15% of patients at 12 months after initiation of ART and at 20% at 18 months. Taking into account a loss to follow-up of 10% per year and 20% failure rate, 83 patients are needed per study arm

Confidential

to detect differences in resistance development as described in literature.

The study is also powered to detect the expected differences in selection of multiple resistance mutations in both arms at month 18 after therapy initiation, derived from a pilot study we performed at Ndlovu Medical Centre.[15] In this study we saw an increase of 2.8 to 4.3 mutations per additional 6-12 months of prolonged therapy. This difference can be detected with 10 patients with therapy failure (59 patients per study arm at an expected failure rate of 20% and 10% loss to follow-up a year).

The third outcome used for power calculation is based on a cross-sectional analysis of a detectable VL >1000 copies/ml at the end of the trial (study month 24). The number of patients with a VL test result above 1000 copies/mL is estimated to be 15% in the control arm and 5% in the intervention arm. Taking into account a loss to follow-up of 10% a year, initial inclusion of 216 patients per arm is required to detect the anticipated difference.

Confidential

7 ETHICAL CONSIDERATIONS

7.1 Regulation statement

This study protocol will be sent to the Research Ethics Committee of the Faculty of Health Sciences, University of Pretoria and to the Ethics Committee of Limpopo province for ethical approval before the start of the study. The ITREMA trial will be registered at the National Health Research Ethics Council (NHREC) and the South African National Clinical Trials Register (SANCTR).

This trial will be conducted in accordance with the ethical principles of:

- World Medical Association Declaration of Helsinki
- ICH GCP guidelines
- Guidelines for Good Practice in the Conduct of Clinical Trials in Human Participants in South Africa.

7.2 Recruitment and consent

7.2.1 Timelines

This trial is planned to run during an initial enrolment period of 18 months and a follow-up period of 2 years. The total study duration will be 3.5 years.

7.2.2 Recruitment procedure

HIV-positive patients over the age of 18 years attending Ndlovu Medical Center will be approached by a health care worker. If patients agree to being informed about the study, they will be screened for inclusion and exclusion criteria (see "3.4 Inclusion criteria" and "3.5 Exclusion criteria") and the informed consent procedure is performed according to "6.2.5 Informed consent". If informed consent is obtained, all other activities described under "4.2.1.1 Baseline study visit" will be performed at the same time point (baseline study visit).

7.2.3 Informed consent

The informed consent procedure will be performed by a research health care worker, in the subjects' home language if necessary. The participant information sheet and informed consent form will be in English. Risks and benefits of study participation will be discussed and all questions will be answered. Written informed consent will be obtained for the study. Patients can give informed consent by signature, thumbprint or ID-mark. In case of patients that are unable to read or write, the health care worker performing the informed consent procedure and an impartial witness will co-sign the consent form with approval of the consenting participant.

Confidential

Participants may refuse to participate in the study or may withdraw consent at any time and this will not affect their routine clinical care. The participant will receive a copy of the informed consent for their records. Another copy of the informed consent will be filed in the patient's record. Patients will also receive a contact number at which they can contact the study clinician in case of queries or other events.

7.2.4 Randomisation procedure

The randomisation procedure takes place between the month 6 and month 9 study visit if randomisation criteria are met (see "4.5 Randomisation criteria"). Randomisation will be performed by allocating participant study numbers into either of two treatment arms using a statistical software package. Patients are informed of the result of randomisation between the month 6 and month 9 study visit.

7.3 Benefits and risks assessment

7.3.1 Potential benefits associated with participation

7.3.1.1 Immediate benefit

Intensified monitoring will allow for early detection of suboptimal adherence, viremia and drug resistance in patients randomized to the intervention arm of this trial. This will result in faster switching of therapeutic regimen in case of drug resistance, avoiding unnecessary switches to second line therapy in case of no resistance, and in more intensive adherence counselling in the case of suboptimal adherence, which are all in the interest of the patient.

7.3.1.2 Long-term benefit

By participation in this study, patients contribute to the development of more efficient methods of HIV-1 treatment monitoring for future patients as well as expansion in knowledge of risk factors and determinants of HIV-1 therapy failure.

7.3.2 Potential risks associated with participation

7.3.2.1 Blood sampling

Minimal additional risk. When blood is drawn, patients might experience slight discomfort or possible inflammation at the site where blood is drawn. A qualified health care worker will draw the blood and explain the process to participants.

7.3.2.2 Drug level monitoring

See above: "6.3.2.1 Blood sampling"

Confidential

7.3.2.3 Questionnaires

Minimal risk. Participants might find certain questions of a sensitive nature. Referral to a social worker for psychosocial support will be available, if needed.

7.4 Incentives

This trial will study the effect of an intervention in a clinical care setting. Study participants will not be given reimbursements for study visits, as these study visits coincide with scheduled follow-up visits at the clinic. Study participants will receive adequate referral to standard medical care in case of medical conditions diagnosed during study follow-up.

Confidential

8 ADMINISTRATIVE ASPECTS, MONITORING AND PUBLICATION

8.1 Handling and storage of data and documents

8.1.1 Data collection

Clinical data will be collected by study health care workers using data collection forms, case report forms and questionnaire forms. Clinical data will be captured into a digital database accessible only by the research team, data monitor and password protected.

8.1.2 Patient confidentiality

Participants will be assigned a unique study identification number at the baseline study visit. Data analysis will be conducted using this number and will not be associated with any personal identifying information. A participant identification code list will be used to link the data to the subject. This participant identification code list should remain on site.

All records with personal identifying information will be accessible only to specific study personnel for the purposes expressly consented to by the study participant. Extra blood sample for storage will be stored anonymously without any personal identifying information, using the study identification number.

8.1.3 Record retention

The site Principal Investigator will retain in a secure manner, complete, accurate and current trial records for a minimum of 15 years after the conclusion of the study. Trial records include administrative documentation, including all reports and correspondence relating to the trial, as well as documentation related to each participant screened and/or enrolled in the trial, including informed consent forms, CRFs, notations of all contacts with the participant and all other source documents. No records will be removed from the site throughout the trial's period of performance.

8.2 Monitoring and Quality Assurance

During or after the clinical trial, the governmental regulatory authorities, local IRB/IEC and/or representatives from the consortium may request access to all trial documents for a research centre audit or inspection.

Confidential

8.3 Amendments

Amendments are changes made to the research protocol and associated study documents after a favourable opinion has been obtained by the Ethics Committee. All amendments will be submitted for approval by the FHS Research Ethics Committee at the University of Pretoria.

8.4 Implementation and dissemination

8.4.1 Implementation

The results of the ITREMA study will be disseminated among key stakeholders. If the proposed intensified monitoring strategy is proven to be cost–effective, informing of decision makers in South Africa and other low- and middle-income settings is the next step. Documentation on the implementation of the intensified monitoring strategy will be freely available to clinics and organisations and will be distributed on demand by South African study partners. This documentation will contain the necessary information to facilitate transfer of the DLM assay and the DBS spotting technique, as well as the intensified monitoring strategy as a whole including DLM-tailored adherence counselling and frequent viral load testing.

8.4.2 Dissemination

The main objective of dissemination of study results is to present the results of the study to other clinics in low- and middle-income countries. The dissemination effort is aimed at medical and scientific peers in the national South African setting and from the international community and the South African NDoH. These stakeholders will be informed on study results in a policy document.

8.5 Interim and end of study reports

The sponsor/investigator will submit a summary of the progress of the trial to the accredited Ethics Committee once a year. Information will be provided on the date of inclusion of the first subject, numbers of subjects included and numbers of subjects that have completed the trial, other problems and amendments.

At the end of this study the Ethics Committee will be informed within a period of 8 weeks. The end of the study is defined as the last patient's last visit. Within one year after the end of the study a final study report with the results of the study, including any publications/abstracts of the study, will be submitted to the Ethics Committee.

Confidential

8.6 Public disclosure and publication policy

Results will be published in peer-reviewed scientific journals and presented at international and local scientific meetings.

Confidential

9 REFERENCES

1. Barth RE, van der Loeff MFS, Schuurman R, Hoepelman AIM, Wensing AMJ. Virological follow-up of adult patients in antiretroviral treatment programmes in sub-Saharan Africa: a systematic review. *Lancet Infect Dis*. 2010;10(3):155–66. doi:10.1016/S1473-3099(09)70328-7.

2. Goldie SJ, Yazdanpanah Y, Losina E, et al. Cost-effectiveness of HIV treatment in resource-poor settings—the case of Côte d'Ivoire. *… Engl J …*. 2006:1141–1153. Available at: http://www.nejm.org/doi/full/10.1056/NEJMsa060247. Accessed August 27, 2014.

3. Bishai D, Colchero A, Durack DT. The cost effectiveness of antiretroviral treatment strategies in resource-limited settings. *AIDS*. 2007;21(10):1333–40. doi:10.1097/QAD.0b013e328137709e.

4. Vijayaraghavan A, Efrusy MB, Mazonson PD, Ebrahim O, Sanne IM, Santas CC. Cost-effectiveness of alternative strategies for initiating and monitoring highly active antiretroviral therapy in the developing world. *J Acquir Immune Defic Syndr*. 2007;46(1):91–100. doi:10.1097/QAI.0b013e3181342564.

5. Phillips AN, Pillay D, Miners AH, Bennett DE, Gilks CF, Lundgren JD. Outcomes from monitoring of patients on antiretroviral therapy in resource-limited settings with viral load, CD4 cell count, or clinical observation alone: a computer simulation model. *Lancet*. 2008;371(9622):1443–51. doi:10.1016/S0140-6736(08)60624-8.

6. Bendavid E, Young S, Katzenstein D, Bayoumi A, Gillian D, Owens D. Cost-effectiveness of HIV monitoring strategies in resource-limited settings: a southern African analysis. *Arch …*. 2008;168(17):1910–1918. Available at: http://archpedi.jamanetwork.com/article.aspx?articleid=773433. Accessed August 27, 2014.

7. Kahn JG, Marseille E, Moore D, Ekwaru P, Kaharuza F, Mermin J. CD4 cell count and viral load monitoring in patients undergoing antiretroviral therapy in Uganda : cost effectiveness study. 2011;6884(November):1–12. doi:10.1136/bmj.d6884.

8. Medina Lara A, Kigozi J, Amurwon J, et al. Cost effectiveness analysis of clinically driven versus routine laboratory monitoring of antiretroviral therapy in Uganda and Zimbabwe. *PLoS One*. 2012;7(4):e33672. doi:10.1371/journal.pone.0033672.

9. Walensky RP, Ciaranello AL, Park J-E, Freedberg K a. Cost-effectiveness of laboratory monitoring in sub-Saharan Africa: a review of the current literature. *Clin Infect Dis*. 2010;51(1):85–92. doi:10.1086/653119.

10. Ford N, Roberts T, Calmy A. Viral load monitoring in resource-limited settings: a medical and public health priority. *AIDS*. 2012;26(13):1719–20. doi:10.1097/QAD.0b013e3283543e2c.

Confidential

11. Hamers RL, Sawyer a W, Tuohy M, Stevens WS, Rinke de Wit TF, Hill AM. Cost-effectiveness of laboratory monitoring for management of HIV treatment in sub-Saharan Africa: a model-based analysis. *AIDS*. 2012;26(13):1663–72. doi:10.1097/QAD.0b013e3283560678.

12. Rawizza HE, Chaplin B, Meloni ST, et al. Immunologic Criteria Are Poor Predictors of Virologic Outcome : Implications for HIV Treatment Monitoring in Resource-Limited Settings. 2011;53:1283–1290. doi:10.1093/cid/cir729.

13. Westley BP, Delong AK, Tray CS, et al. Prediction of Treatment Failure Using 2010 World Health Organization Guidelines Is Associated With High Misclassification Rates and Drug Resistance Among HIV-Infected Cambodian Children. 2012;55:432–440. doi:10.1093/cid/cis433.

14. Kantor R, Diero L, Delong A, et al. Misclassification of First-Line Antiretroviral Treatment Failure Based on Immunological Monitoring of HIV Infection in Resource-Limited Settings. 2009;02906. doi:10.1086/600396.

15. Barth RE, Aitken SC, Tempelman H, et al. Accumulation of drug resistance and loss of therapeutic options precede commonly used criteria for treatment failure in HIV-1 subtype-C-infected patients. *Antivir Ther*. 2012;17(2):377–86. doi:10.3851/IMP2010.

16. World Health Organization. Consolidated guidelines on the use of antiretroviral drugs for treating and preventing HIV infection. 2013. 2013. Available at: http://scholar.google.com/scholar?hl=en&btnG=Search&q=intitle:Consolidated+Guidelines+on+the+use+of+Antiretroviral+Drugs+for+treating+and+preventing+HIV+Infection#2. Accessed April 30, 2014.

17. National Department of Health (South Africa). National consolidated guidelines for the prevention of mother-to-child transmission of HIV (PMTCT) and the management of HIV in children, adolescents and adults. 2014;(December):2–119.

18. Meintjes G, Maartens G, Boulle A, et al. Guidelines for antiretroviral therapy in adults. 2012;13(3):36–45. doi:10.7196/SAJHIVMED.862.

19. Sigaloff KCE, Ramatsebe T, Viana R, de Wit TFR, Wallis CL, Stevens WS. Accumulation of HIV drug resistance mutations in patients failing first-line antiretroviral treatment in South Africa. *AIDS Res Hum Retroviruses*. 2012;28(2):171–5. doi:10.1089/aid.2011.0136.

20. Kantor R, Zijenah L, Shafer R, et al. HIV-1 subtype C reverse transcriptase and protease genotypes in Zimbabwean patients failing antiretroviral therapy. *AIDS Res Hum Retroviruses*. 2002;18(18):1407–1413. doi:10.1089/088922202320935483.HIV-1.

21. Weidle PJ, Malamba S, Mwebaze R, et al. Assessment of a pilot antiretroviral drug therapy programme in Uganda: patients' response, survival, and drug resistance. *Lancet*. 2002;360(9326):34–40. doi:10.1016/S0140-6736(02)09330-3.

Confidential

22. Marconi VC, Sunpath H, Lu Z, et al. Prevalence of HIV-1 drug resistance after failure of a first highly active antiretroviral therapy regimen in KwaZulu Natal, South Africa. *Clin Infect Dis*. 2008;46(10):1589–97. doi:10.1086/587109.

23. Murphy RA, Sunpath H, Lu Z, et al. Outcomes after virologic failure of first-line ART in South Africa. *AIDS*. 2010;24(7):1007–1012. Available at: http://www.ncbi.nlm.nih.gov/pmc/articles/PMC2902159/. Accessed February 21, 2015.

24. Zyl GU Van, Merwe L Van Der, Claassen M, Zeier M, Preiser W. Antiretroviral Resistance Patterns and Factors Associated With Resistance in Adult Patients Failing NNRTI-Based Regimens in the Western Cape, South Africa. *J Med Virol*. 2011;83(June):1764–1769. doi:10.1002/jmv.

25. Van Zyl GU, Liu TF, Claassen M, et al. Trends in Genotypic HIV-1 Antiretroviral Resistance between 2006 and 2012 in South African Patients Receiving First- and Second-Line Antiretroviral Treatment Regimens. *PLoS One*. 2013;8(6):e67188. doi:10.1371/journal.pone.0067188.

26. Manasa J, Lessells RJ, Skingsley A, et al. High-levels of acquired drug resistance in adult patients failing first-line antiretroviral therapy in a rural HIV treatment programme in KwaZulu-Natal, South Africa. *PLoS One*. 2013;8(8):e72152. doi:10.1371/journal.pone.0072152.

27. Aitken S, Slabbert M, Schrooders P, Tempelman H, Schuurman R, Wensing A. HIV-1 resistance testing on dried blood spots enables individual patient management in arural South-African setting. In: *Towards virological monitoring of HIV-1 drug resistance in resource-limited settings.*; 2013.

28. Aghokeng AF, Monleau M, Eymard-Duvernay S, et al. Extraordinary heterogeneity of virological outcomes in patients receiving highly antiretroviral therapy and monitored with the World Health Organization public health approach in sub-saharan Africa and southeast Asia. *Clin Infect Dis*. 2014;58(1):99–109. doi:10.1093/cid/cit627.

29. Asboe D, Aitken C, Boffito M, et al. British HIV Association guidelines for the routine investigation and monitoring of adult HIV-1-infected individuals 2011. *HIV Med*. 2012;13(1):1–44. doi:10.1111/j.1468-1293.2011.00971.x.

30. Panel on Antiretroviral Guidelines for Adults and Adolescents. Guidelines for the use of antiretroviral agents in HIV-infected adults and adolescents. *Dep Heal Hum Serv*. 2013. Available at: http://aidsinfo.nih.gov/ContentFiles/Adultand.

31. Kredo T, Van der Walt J, Siegfried N, Cohen K. Therapeutic drug monitoring of antiretrovirals for people with HIV. *Cochrane database Syst Rev*. 2009;(3). Available at: http://onlinelibrary.wiley.com/doi/10.1002/14651858.CD007268.pub2/full. Accessed February 15, 2015.

32. Tanser F, Bärnighausen T, Grapsa E, Zaidi J, Newell M-L. High coverage of ART associated with decline in risk of HIV acquisition in rural KwaZulu-Natal, South Africa. *Science*. 2013;339(6122):966–71. doi:10.1126/science.1228160.

Confidential

33. World Health Organization. WHO HIV drug resistance report. 2012.

34. Saenz R a, Bonhoeffer S. Nested model reveals potential amplification of an HIV epidemic due to drug resistance. *Epidemics*. 2013;5(1):34–43. doi:10.1016/j.epidem.2012.11.002.

35. L'homme RF a, Muro EP, Droste J a H, et al. Therapeutic drug monitoring of nevirapine in resource-limited settings. *Clin Infect Dis*. 2008;47(10):1339–44. doi:10.1086/592694.

36. Holland D, Espina-Quinto R, Stefanski E, Moon B, Valdez J, Connor J. Rapid automated immunoassay for therapeutic drug monitoring of nevirapine using ARK NVP-test: method validation, application and comparison with HPLC method (abstract). In: *ICCAC*.; 2005:A34.

37. Salado-Rasmussen K, Theilgaard ZP, Chiduo M, Pedersen C, Gerstoft J, Katzenstein TL. Good performance of an immunoassay based method for nevirapine measurements in human breast milk. *Clin Chem Lab Med*. 2011;49(7):1171–5. doi:10.1515/CCLM.2011.184.

38. Abdissa A, Wiesner L, Mcilleron H, Friis H. Short report Evaluation of an immunoassay for determination of plasma efavirenz concentrations in resource-limited settings. *J Int AIDS Soc*. 2014:1–5.

39. George L, Muro EP, Ndaro A, Dolmans W, Burger DM, Kisanga ER. Nevirapine concentrations in saliva measured by thin layer chromatography and self-reported adherence in patients on antiretroviral therapy at kilimanjaro christian medical centre, Tanzania. *Ther Drug Monit*. 2014;36(3):366–70. doi:10.1097/FTD.0000000000000005.

40. Aitken SC, Bronze M, Wallis CL, et al. A pragmatic approach to HIV-1 drug resistance determination in resource-limited settings by use of a novel genotyping assay targeting the reverse transcriptase-encoding region only. *J Clin Microbiol*. 2013;51(6):1757–61. doi:10.1128/JCM.00118-13.

41. Mermin J, Granich R. Economics of monitoring antiretroviral therapy. *Lancet Infect Dis*. 2013;13(7):560–1. doi:10.1016/S1473-3099(13)70105-1.

42. Rowley CF. Developments in CD4 and viral load monitoring in resource-limited settings. *Clin Infect Dis*. 2014;58(3):407–12. doi:10.1093/cid/cit733.

43. Krentz HB, Ko K, Beckthold B, Gill MJ. The cost of antiretroviral drug resistance in HIV-positive patients. *Antivir Ther*. 2014;19(4):341–8. doi:10.3851/IMP2709.

44. Levison JH, Wood R, Scott C a, et al. The clinical and economic impact of genotype testing at first-line antiretroviral therapy failure for HIV-infected patients in South Africa. *Clin Infect Dis*. 2013;56(4):587–97. doi:10.1093/cid/cis887.

Confidential

45. Swenson LC, Min JE, Woods CK, et al. HIV drug resistance detected during low-level viraemia is associated with subsequent virologic failure. *AIDS*. 2014;(November 2013):1125–1134. doi:10.1097/QAD.0000000000000203.

46. Aitken SC, Kliphuis A, Bronze M, et al. Development and evaluation of an affordable real-time qualitative assay for determining HIV-1 virological failure in plasma and dried blood spots. *J Clin Microbiol*. 2013;51(6):1899–905. doi:10.1128/JCM.03305-12.

47. Pitman R, Fisman D, Zaric GS, et al. Dynamic transmission modeling: a report of the ISPOR-SMDM Modeling Good Research Practices Task Force--5. *Value Health*. 2012;15(6):828–34. doi:10.1016/j.jval.2012.06.011.

48. National Department of Health. *The 2011 National Antenatal Sentinel HIV & Syphilis Prevalence Survey in South Africa*.

49. Sunpath H, Wu B, Gordon M. High rate of K65R for ART naïve patients with subtype C HIV infection failing a TDF-containing first-line regimen in South Africa. *AIDS (London, …*. 2012;26(13):1679–1684. doi:10.1097/QAD.0b013e328356886d.High.

50. Theys K, Vercauteren J, Snoeck J, et al. HIV-1 subtype is an independent predictor of reverse transcriptase mutation K65R in HIV-1 patients treated with combination antiretroviral therapy including tenofovir. *Antimicrob Agents Chemother*. 2013;57(2):1053–6. doi:10.1128/AAC.01668-12.

Confidential
